# Role of Phytochromes in Red Light-Regulated Alternative Splicing in *Arabidopsis thaliana*: Impactful but Not Indispensable

**DOI:** 10.3390/cells12202447

**Published:** 2023-10-13

**Authors:** Daniel Alejandro Careno, Constanza Helena Assaf, Eline Dieuwerke Catharina Eggermont, Micaela Canelo, Pablo Diego Cerdán, Marcelo Javier Yanovsky

**Affiliations:** 1 Fundación Instituto Leloir, Instituto de Investigaciones Bioquímicas de Buenos Aires–Consejo Nacional de Investigaciones Científicas y Técnicas (IIBBA-CONICET), Buenos Aires C1405BWE, Argentina; cassaf@leloir.org.ar (C.H.A.); eline.eggermont@wur.nl (E.D.C.E.); mcanelo@leloir.org.ar (M.C.); pcerdan@leloir.org.ar (P.D.C.); 2 Departamento de Fisiología, Biología Molecular y Celular, Facultad de Ciencias Exactas y Naturales, Universidad de Buenos Aires, Buenos Aires C1428EGA, Argentina; 3 Plant-Environment Signaling Group, Department of Biology, Utrecht University, 3584 CH Utrecht, The Netherlands

**Keywords:** alternative splicing, phytochrome, light signaling, Arabidopsis

## Abstract

Light is both the main source of energy and a key environmental signal for plants. It regulates not only gene expression but also the tightly related processes of splicing and alternative splicing (AS). Two main pathways have been proposed to link light sensing with the splicing machinery. One occurs through a photosynthesis-related signal, and the other is mediated by photosensory proteins, such as red light-sensing phytochromes. Here, we evaluated the relative contribution of each of these pathways by performing a transcriptome-wide analysis of light regulation of AS in plants that do not express any functional phytochrome (*phyQ*). We found that an acute 2-h red-light pulse in the middle of the night induces changes in the splicing patterns of 483 genes in wild-type plants. Approximately 30% of these genes also showed strong light regulation of splicing patterns in *phyQ* mutant plants, revealing that phytochromes are important but not essential for the regulation of AS by R light. We then performed a meta-analysis of related transcriptomic datasets and found that different light regulatory pathways can have overlapping targets in terms of AS regulation. All the evidence suggests that AS is regulated simultaneously by various light signaling pathways, and the relative contribution of each pathway is highly dependent on the plant developmental stage.

## 1. Introduction

Alternative splicing (AS) is a gene expression regulatory mechanism through which a single gene can generate numerous distinct transcripts. These different transcripts may encode various protein isoforms or influence mRNA stability via nonsense-mediated decay (NMD) or microRNA regulation [1]. The specific mature mRNA produced relies on the interaction between trans-acting regulators recruited by cis-acting elements present in the pre-mRNA. This interaction determines splice-site selection and the assembly of the spliceosome, a ribonucleoprotein complex that catalyzes intron removal and exon ligation [2]. Different AS isoforms can arise from intron retention (IR), exon skipping (ES), or the utilization of alternative 5′ donor and/or 3′ acceptor splice sites (Alt5’ss and Alt3’ss). In plants, the most prevalent AS event is IR [1].

Determining the relative importance of the different isoforms resulting from AS is challenging. Nonetheless, examples exist that illustrate their roles in plant physiology. For instance, the negative regulator of photomorphogenesis, *SPA1-RELATED 3* (*SPA3*) has both functional and non-functional AS isoforms, which may differentially contribute to the de-etiolation process [3]. Furthermore, the circadian clock gene *PSEUDO RESPONSE REGULATOR 9* (*PRR9*) presents two AS isoforms that oscillate with distinct phases, likely necessary for maintaining proper clock pace [4]. It has also been demonstrated that elevated temperatures promote the accumulation of an NMD-susceptible AS isoform of the flowering repressor *FLOWERING LOCUS M* (*FLM*), advancing the transition from vegetative growth to the reproductive stage [5].

In plants, light constitutes a major environmental signal regulating splicing patterns [6]. Two mechanisms by which light controls AS have been described. First, a retrograde signal from the chloroplast linked to the redox state of the plastoquinone pool has been shown to regulate the splicing patterns of splicing factor transcripts, such as *RS31*, *U2AF65,* and *SR30* [7]. This retrograde signal enhances RNA Pol II elongation speed, favoring the selection of strong splice sites, while darkness slows RNA Pol II elongation, promoting the use of weak splice sites [8]. In roots, AS is regulated by sugars generated during photosynthesis in a TARGET OF RAPAMYCIN (TOR)-dependent manner [9].

Second, AS is regulated by sensory photoreceptors. Plants have a variety of photosensory photoreceptors that allow them to sense and respond to different wavelengths of light [10]. To a great extent, these photoreceptors act by modulating gene expression at the transcriptional level [10]. In addition, recent research has unveiled a role for the blue-light-sensing photoreceptor cryptochrome 2 (cry2) in the control of gene expression through the modulation of AS. Cry2 modulates the binding activity of the RNA binding protein CRYPTOCHROME INTERACTING SPLICING FACTOR 1 (CIS1), thereby influencing the splicing of *FLM* pre-mRNA and consequently the floral transition [5]. Other photoreceptors involved in the regulation of AS are red (R) and far red (FR) light-sensing phytochromes, which are pivotal in controlling a plethora of physiological processes, such as germination, de-etiolation, shade avoidance syndrome, circadian clock entrainment, and flowering. The *Arabidopsis thaliana* genome encodes five phytochromes (phyA-E), with phyA and phyB being more important and extensively characterized than phyC, phyD, and phyE [11].

R light induces a structural change in phytochrome proteins, favoring the transition from the inactive Pr conformation to the active Pfr conformation. Pr is cytoplasmic, while Pfr primarily resides in the cell nucleus. During etiolated growth, phytochromes remain in their Pr conformation, while multiple transcription factors in the nucleus induce the etiolation growth program. When dark-grown plants perceive R light, phytochromes turn to their Pfr conformation, translocate to the nucleus, and physically interact and inhibit the action of the etiolated growth-promoting factors PHYTOCHROME-INTERACTING FACTORs (PIFs), CONSTITUTIVE PHOTOMORPHOGENIC1 (COP1), and SUPPRESSOR OF PHYA-105 (SPA), thus initiating the light response [12]. The molecular mechanism behind the shade avoidance response shares some degree of similarity with the de-etiolation mechanism. Shade, i.e., a low R to FR-light ratio, promotes the photoconversion of Pfr to Pr. Consequently, the activity of COP1, SPA, and the PIF family is de-repressed and the typical shade-avoidance response takes place, which includes elongation of stems and petioles and increased apical dominance [12]. Additionally, phytochromes, especially phyB, help integrate environmental light signals into the circadian clock. It has been shown that phyB interacts directly with several clock components in a light-dependent manner [13,14]. Additionally, the null mutants *phyA*, *phyB,* and *phyA phyB* display longer periods than WT plants [15,16]. With respect to flowering, phyA is needed for sensing extensions in the photoperiod, while phyB and phyE regulate photoperiodic flowering at 22 °C and 16 °C, respectively, by acting upstream of FLOWERING LOCUS T (FT) [17,18,19].

It has been reported that phyB also interacts directly and indirectly with multiple splicing factors. SPLICING FACTOR FOR PHYTOCHROME SIGNALING (SFPS) binds to phyB, and both proteins co-localize in the nucleus [20]. SFPS also interacts with two other splicing regulators: REDUCED RED-LIGHT RESPONSES IN CRY1CRY2 BACKGROUND1 (RRC1) and SUPPRESSOR-OF-WHITE-APRICOT/SURP RNA-BINDING DOMAIN-CONTAINING PROTEIN1 (SWAP1) [21,22]. SFPS, RRC1, and SWAP1 are necessary for the correct light-regulated splicing of multiple pre-mRNAs of genes involved in photomorphogenesis [20,21,22,23]. While RCC1 and phyB may co-localize, recent evidence suggests that their activities may be, in part, spatially uncoupled. PhyB senses R light in epidermal tissue and initiates a signaling cascade that ultimately involves RCC1 in endodermal tissue, regulating the splicing pattern of several genes, including the clock genes *PRR7*, *TOC1*, *JMJD30,* and *ELF3* [24]. The loss-of-function mutants *sfps*, *rcc1,* and *swap1* exhibit phenotypes similar to those observed in the *phyB* mutant during de-etiolation under R light. Conversely, the splicing-related proteins PRE-mRNA-PROCESSING PROTEIN 40 (PRP40) and SWELLMAP 2 (SMP2) have also been implicated in the R-light signaling pathway but have a suppressor role in photomorphogenesis [25,26]. Specifically, SMP2 physically interacts with phyB and regulates the splicing of the core clock gene *RVE8* [26]. As expected, the *phyA phyB* double mutant displays strong alterations in the splicing landscape during photomorphogenesis [3].

In a previous study, we demonstrated that an acute white-light pulse treatment in the middle of the night alters the splicing patterns of hundreds of genes [27]. However, the extent to which each of the aforementioned pathways contributes to regulating AS in light-grown plants remains unknown. Additionally, even though the role of phytochromes in the AS regulatory network has been studied in etiolated seedlings, their role in controlling AS in light-grown plants has yet to be analyzed in detail. The quintuple phytochrome mutant (*phyABCDE*, hereafter *phyQ*) has been extensively characterized at the physiological and molecular levels [28,29,30]. *phyQ* mutant plants are completely insensitive to R light for the induction of photomorphogenic responses in etiolated seedlings, behaving as dark-grown plants under this condition [28,30]. Furthermore, they are nearly blind to an acute 2-h R-light pulse that triggers transcriptional reprogramming in etiolated wild-type (WT) seedlings [30]. In contrast, *phyQ* mutant plants de-etiolate almost normally upon exposure to blue light, which activates other families of photosensory photoreceptors [28,30]. In light-grown plants, phytochromes are critical for the regulation of growth and development [28,30], and this is largely mediated through their effect on the plant transcriptome [30,31]. In this study, we sought to quantify the contribution of phytochromes to the regulation of the AS landscape in light-grown Arabidopsis plants. To investigate this, we compared splicing patterns between WT and *phyQ* mutant plants in response to an acute R-light treatment in Arabidopsis seedlings that were previously de-etiolated under white-light conditions.

## 2. Materials and Methods

### 2.1. Plant Material and Growth Conditions

All experiments were performed with the Arabidopsis Columbia (Col-0) ecotype and the previously described quintuple phytochrome mutant [28,29]. The *phyQ* quintuple mutant was obtained by crossing the following mutant lines: *phyA-211* [18]; *phyB-9* [32]; *phyC-2* [33]; *phyD-201* (Salk_027956) and *phyE-201* (Salk_040131) [28,34]. The primers used for genotyping are detailed in Supplementary Table S12.

**Figure 1:**
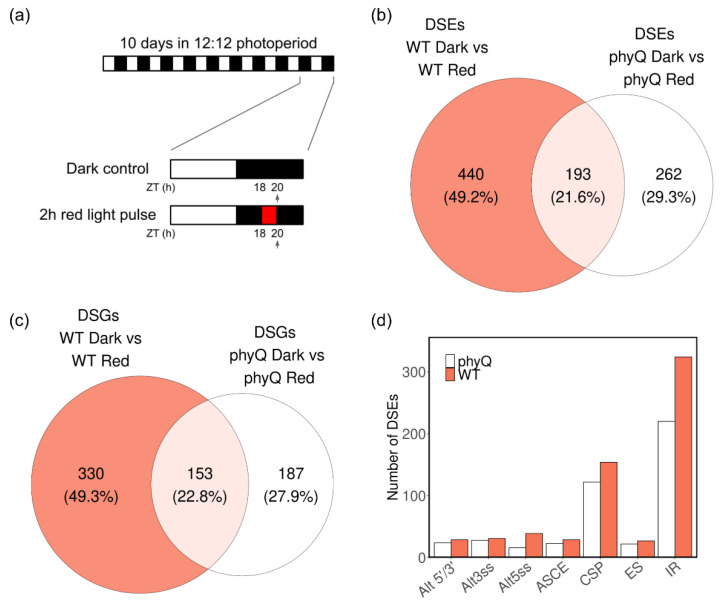
Phytochromes are needed to fine-tune the AS landscape. (**a**) Experimental setup. Plants were grown under a 12:12 photoperiod for 10 days. Then, they were either kept in darkness or treated with a 2-h R-light pulse in the middle of the night. The arrows indicate the harvesting time. (**b**) Differentially spliced events (DSEs) and (**c**) differentially spliced genes (DSGs) upon R-light treatment in WT and *phyQ* seedlings. (**d**) Amount of each type of AS event in the DSEs between WT dark vs. WT R, and *phyQ* dark vs. *phyQ* R. Alt, alternative (5′ and/or 3′) splicing site; ES: exon skipping; IR, intron retention; ASCE, AS affecting a consensus exon; CSP, complex splicing pattern.

The seeds were sterilized with chlorine in the vapor phase. For the night-break experiment, sterilized WT and *phyQ* mutant seeds were suspended in 100 μM GA 4 + 7 (Duchefa Biochemie), stratified for 3 days at 4 °C, and pipetted onto plates with Murashige and Skoog (MS) salt media and 0.8% plant agar (Duchefa Biochemie). Then, plants were grown under 12-h light:12-h dark cycles at 22 °C (50 µmol m^−2^ s^−1^ of white light provided by fluorescent tubes). After 10 days, the seedlings were irradiated in the middle of the night (ZT18) with a 2-h R-light pulse or kept in darkness as a control. The R-light pulse was provided with light-emitting diodes (Kingbright Super Bright LEDs, L934-SRC, λ = 660 ± 20 nm). A brief summary of the experimental protocol is described in Figure 1a. The experiment was performed in triplicate, and in all cases, samples of the light-treated and dark control groups were harvested at the same time and flash frozen in liquid nitrogen.

### 2.2. High-Throughput RNA Sequencing

Library generation and sequencing were performed by Novogene (USA). Briefly, messenger RNA was purified from total RNA using poly-T oligo-attached magnetic beads. After fragmentation, first-strand cDNA was synthesized using random hexamer primers followed by second-strand cDNA synthesis. Next, adapters were ligated, and fragments were selected by size and amplified. Samples were sequenced on the Illumina NovaSeq 6000 platform, providing 150-bp paired-end reads. On average, 61 million 150-nt paired-end reads were obtained for each sample (Supplementary Table S1).

### 2.3. Processing of RNA Sequencing Reads

Sequence reads were mapped to the Arabidopsis TAIR10 [35] genome using STAR 2.7.0 [36] with the following parameters: *twopassMode* Basic, *outFilterMultimapNmax* 2, *outFilterType* BySJout, *outSJfilterReads* Unique, *sjdbOverhang* 149, *alignSJoverhangMin* 6, *alignSJDBoverhangMin* 3, *alignIntronMin* 20 and *alignIntronMax* 5000. The average percentage of uniquely mapped reads was 86.75% (Supplementary Table S1).

### 2.4. Differential Splicing Analysis

Differential splicing events (DSEs) were evaluated using the ASpli package version 2.10.0 in R 4.1.1 [37]. First, genes with fewer than 50 mapping reads and read densities lower than 0.05 were filtered. Additionally, bins with fewer than 25 mapping reads were discarded for the following analysis. Genes were considered differentially expressed between two conditions if |log_2_(FC)| > log_2_(3) with an associated FDR < 0.01. For splicing analysis, PSI (percent of inclusion) or PIR (percent of IR) were calculated. A bin was considered to be differentially spliced if the bin coverage |log_2_(FC)| > log_2_(3) with an associated FDR < 0.05 or ΔPSI/PIR > 0.1 with an associated FDR < 0.05. For the meta-analysis, we reanalyzed the datasets with these same parameters whenever data from independent biological replicates were publicly available. Principal component analysis was performed on normalized reads by DESeq2′s median of ratios [38].

### 2.5. Differential Gene Expression Analysis

Differential gene expression analysis was also performed using ASpli, with the same expression filters used for the differential splicing analysis. Simple contrasts between the following experimental conditions were conducted: WT dark vs. WT R (Supplementary Table S4), *phyQ* dark vs. *phyQ* R (Supplementary Table S5), WT dark vs. *phyQ* dark, and WT R vs. *phyQ* R. Additionally, the ASpli formula approach was utilized to estimate through a generalized linear model the effect of the genotype, the light treatment, and the interaction between both. Genes with an FDR < 0.01 and |log_2_(FC)| > log_2_(1) were considered to be differentially expressed (Supplementary Tables S4 and S5). For expression plots, raw counts were normalized with DESeq2 [38]. For each condition, 3 biological replicates were analyzed.

### 2.6. GO Analysis

The assignment of GO terms for the differentially expressed genes (DEGs) and differentially spliced genes (DSGs) datasets was obtained using the Bioconductor packages topGO version 2.44.0 and org.At.tair.db version 3.13.0. An enrichment test was performed for the biological process category. *p* values were obtained using Fisher’s exact test. The enrichment factor (EF) was estimated as the ratio between the proportions of genes associated with a particular GO category present in the dataset under analysis relative to the proportion of the number of genes in this category in the whole genome that passed the expression filters.

### 2.7. RNA Isolation and AS Event Validation by PCR Analysis

For AS validation, three biological replicates of Arabidopsis plants from the Col-0 accession and *phyQ* mutants were sown on MS-agar plates, cold stratified in darkness for 3 days, and grown for 10 days under 12-h light:12-h dark conditions. Plants were subjected to a 2-h light pulse at ZT18 on the 11th day or kept under dark conditions. Total RNA was extracted with a Spectrum Plant Total RNA Kit (Sigma–Aldrich, St. Louis, MO, USA) following the manufacturer’s protocols. To estimate the concentration and quality of the samples, a NanoDrop 2000c (Thermo Scientific, Waltham, MA, USA) and agarose gel electrophoresis were used. One microgram of RNA was treated with RQ1 RNase-Free DNase (Promega, Madison, WI, USA) and subjected to retro-transcription with Super Script III Reverse Transcriptase (SSIII RT) (Thermo Fisher Scientific, Waltham, MA, USA) and oligo-dT according to the manufacturer’s instructions. Amplification of cDNA was carried out for 30 cycles to measure the relative abundance of the isoforms at the linear phase of amplification and was performed using 1.5 U of Taq polymerase (Invitrogen, Carlsbad, CA, USA). The primers used for amplification are detailed in Supplementary Table S12. RT–PCR products were electrophoresed in 2 or 3% (*w*/*v*) agarose and detected by SYBR Green.

## 3. Results

### 3.1. Red Light Has a Strong Effect on AS in phyQ Mutants

To identify specific AS events regulated by R light and/or phytochromes, we took advantage of the quintuple phytochrome mutant *phyQ*. This mutant is unable to undergo R-light-induced photomorphogenesis and is almost blind to a 2-h R-light treatment that induces large transcriptional changes in WT etiolated seedlings [29]. We conducted a night-break experiment exposing WT and *phyQ* mutant seedlings to a 2-h R-light pulse in the middle of the night (from ZT = 18 h to 20 h) (Figure 1a). Subsequently, we performed RNA high-throughput sequencing on the harvested samples. Principal component analysis revealed a high similarity between biological replicates (Supplementary Figure S1). Notably, the first principal coordinate (PC1) accounted for 54% of the variance, distinguishing R-light-treated samples from control samples, while PC2 explained 24% of the variance, distinguishing the WT from *phyQ* samples. This observation suggests that, in this particular experimental setup, the impact of light treatment on the transcriptome was more significant than that of mutations in all members of the phytochrome gene family.

The analysis of AS identified 633 events regulated by R light in WT plants, corresponding to 483 genes (Figure 1b,c; Supplementary Table S2). Among the 633 differentially spliced events (DSEs), 193 were also differentially spliced in the *phyQ* mutant (Supplementary Table S3). Additionally, 262 DSEs were exclusively observed in *phyQ* mutant plants (Figure 1b). Both WT and *phyQ* plants exhibited comparable proportions of the different types of AS events that were regulated by R light, with IR being the most prevalent type (Figure 1d). A total of 76% and 82% of the DSEs in WT and *phyQ*, respectively, were supported by junction evidence (Supplementary Figure S2).

**Figure 2:**
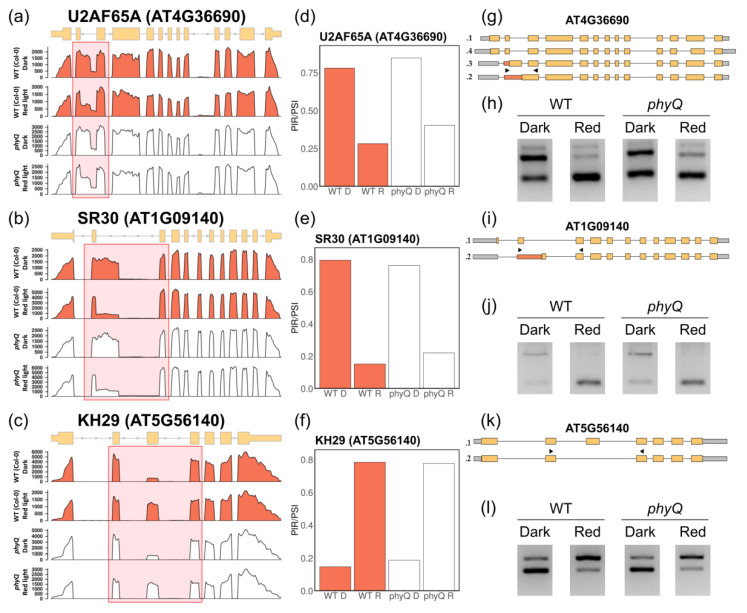
Representative light-regulated but phytochrome-independent AS events. Coverage plot and corresponding gene models of (**a**) *U2AF^64^* (AT4G36690), (**b**) *SR30* (AT1G09140), and (**c**) *KH29* (AT5G56140). The coverage plots were generated by merging data from three distinct biological replicates. (**d**–**f**) Mean percentage spliced-in (PSI) values for each gene across all experimental conditions. (**g**,**i**,**k**) Gene models of the different isoforms annotated for each gene. Boxes represent exons, lines represent introns, and arrows represent the positions of the primers used for validation. (**h**,**j**,**l**) Validation of differentially spliced events (DSEs) through agarose gel electrophoresis of RT–PCR products.

The DSEs responding to R light in both WT and *phyQ* were classified as phytochrome-independent light-regulated events. Notably, some of these events, for instance, those associated with the splicing-related factors *UF2A^64^*, *SR30,* and *KH29*, have previously been reported to be regulated by light under different growth conditions (Figure 2a–c). The PSI for *UF2A^64^* and *SR30* decreased upon R-light treatment, showing an increase in the use of Alt3’ss, while in *KH29*, the third alternative exon was more included (Figure 2d–f). All these AS events were validated by RT–PCR (Figure 2g–l; Supplementary Figure S3). The alternative isoforms introduce an early stop codon in all three genes (Supplementary Figure S4). These results indicate that a large proportion of the R-light-induced changes in the splicing landscape occur independently of the phytochromes, most likely responding to a signal derived from the photosynthetic process.

### 3.2. Phytochromes Are Needed for Splicing of a Subset of Genes

**Figure 3:**
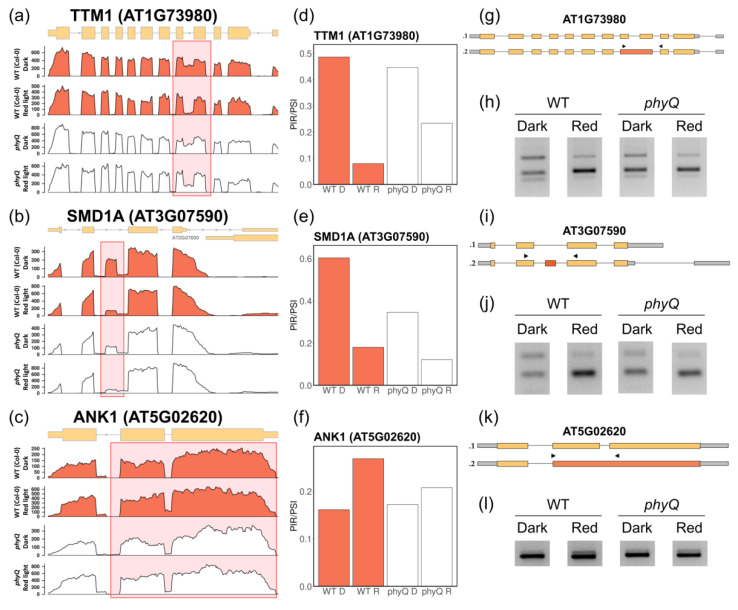
Representative light and phytochrome-regulated splicing events. Coverage plot and corresponding gene models of (**a**) *TTM1* (AT1G73980), (**b**) *SMD1a* (AT3G07590), and (**c**) *ANK1* (AT5G02620). The coverage plots were generated by merging data from three independent biological replicates. (**d**–**f**) Mean percentage spliced-in (PSI) or mean percentage intron retention (PIR) values for each gene across all experimental conditions. (**g**,**i**,**k**) Gene models of the different isoforms annotated for each gene. Boxes represent exons, lines represent introns, and arrows represent the positions of the primers used for validation. (**h**,**j**,**l**) Validation of differentially spliced events (DSEs) through agarose gel electrophoresis of RT–PCR products.

Among the 440 DSEs unique to the WT, a subset showed some degree of dependence on phytochromes (Figure 3a–c). These genes displayed a weaker response to the R-light pulse, as indicated by the smaller dPSI/PIR values between *phyQ* with or without the night-break treatment (Figure 3d–f). Validation of these DSEs was carried out through RT–PCR (Figure 3g–l; Supplementary Figure S5). TTM1 is an organophosphate hydrolase associated with senescence [39]. An IR event regulated by phytochromes leads to a frameshift that causes an early stop codon (Supplementary Figure S6a). Similarly, SMD1a, a component of the Smith (Sm) complex responsible for various steps in RNA metabolism [40], undergoes light-regulated ES through phytochromes, leading to an early stop codon (Supplementary Figure S6b). In turn, the two isoforms of ANK1 resulting from AS encode presumably functional proteins—one with 534 aa and a longer variant with an additional 13 amino acids due to an IR event (Supplementary Figure S6c). A domain scan analysis using the online tool ScanProsite revealed that the longer isoform possesses 5 ankyrin repeat region domains, while the shorter isoform has 6.

We proceeded to investigate the distribution of ΔPIR/PSI values within each genotype. The DSEs responding to the R-light treatment in both genotypes exhibited similar ΔPIR/PSI values for any given event (slope = 0.90, R^2^ = 0.89) (Supplementary Figure S7a). Conversely, the DSEs unique to WT tended to show greater ΔPIR/PSI values in WT than in *phyQ* (slope = 0.26, R^2^ = 0.52). Interestingly, the DSEs exclusively present in *phyQ* displayed rather similar ΔPIR/PSI values in both genotypes (slope = 0.98, R^2^ = 0.53). To further assess the impact of phytochromes on AS efficiency, we calculated ΔΔPIR/PSI as |ΔPIR/PSI*_WT_* − ΔPIR/PSI*_phyQ_*| for each event with junction support (Supplementary Figure S7b). As expected, the DSEs regulated by light in both genotypes exhibited a distribution of ΔΔPIR/PSI with relatively low magnitudes, displaying a slightly higher median value for DSEs where ΔPIR/PSI_WT_ < ΔPIR/PSI*_phyQ_*, indicating a reduced impact of the R-light treatment on AS in *phyQ* mutant plants. Notably, DSEs exclusively identified in WT plants showed a pronounced disparity between the median values of both genotypes. However, when comparing DSEs only detected in *phyQ* mutants, the difference in median values was smaller, revealing a greater overlap in distributions. One plausible explanation is that the DSEs exclusively found in *phyQ* have similar ΔPIR/PSI values in WT plants, but in the latter, they do not reach a small enough FDR to be classified as differentially regulated by light. Collectively, these results underscore the requirement of phytochromes for the proper AS of a specific subset of genes in light-grown plants.

### 3.3. Phytochromes Regulate the Expression of Splicing Factors

**Figure 4:**
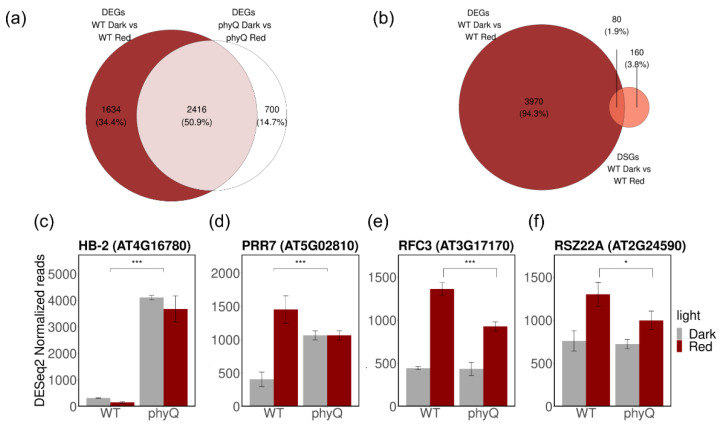
The effect of phytochromes on gene expression. (**a**) Overlap between differentially expressed genes (DEGs) upon an acute R-light pulse in WT and *phyQ*. (**b**) Overlap between DEGs and DSGs in WT. (**c**–**f**) Normalized read count values of (**c**) *HB-2* (AT4G16780), (**d**) *PRR7* (AT5G02810), (**f**) *RSZ22A* (AT2G24590), and (**e**) *RFC3* (AT3G17170). Statistical analysis was performed using ASpli. For *HB-2* and *PRR7*, the statistical significance of the effect of genotype is shown. For *RFC3* and *RSZ22a*, the statistical significance of the contrast between WT R and *phyQ* R is shown. The mean value and standard deviation of three independent biological replicates are shown. *: FDR < 0.05, ***: FDR < 0.001.

Then, we explored changes in gene expression upon R-light treatment and identified 1624 genes differentially expressed in WT plants, while only 1068 DEGs were detected in *phyQ* mutants (Figure 4a; Supplementary Tables S4 and S5). Most of the genes that underwent AS did not change their expression (Figure 4b). As in previous works, there is a small overlap between DEGs and DSGs between WT and mutants of the R- and blue-light signaling pathways [5,24].

It has been reported that the transcript levels of *AtHB-2* and *PIL1* are low in dark and/or white-light conditions in light-grown plants and increase upon FR exposure in WT plants, while in *phy* mutants, the expression levels are always high [41]. This was corroborated in our dataset, confirming that the transcriptome of *phyQ* mutant plants behaved as expected (Figure 3c; Supplementary Figure S8a). Additionally, the expression of the clock gene *PRR7* was upregulated upon R-light treatment in WT plants, while it was unchanged in *pyhQ* mutant seedlings (Figure 3d). This response is consistent with the previously reported function of *PRR7* as a signaling component acting downstream of phytochrome photoreceptors [42].

Some splicing-related genes displayed an altered response to R light in *phyQ* (Figure 3e,f; Supplementary Figure S8b,c). The SR family member *RSZ22a* showed reduced changes in expression in response to R light in *phyQ* mutant seedlings (WT R vs. *phyQ* R log_2_(FC) = 0.35). The function of RSZ22 and its close homologs remains unknown in Arabidopsis [43]. *RFC3* showed a similar tendency (WT R vs. *phyQ* R log_2_(FC) = 0.52) (Figure 3e). The RFC3 protein interacts with plastid splicing factors [44]. Additionally, *RS41* and *U2AF^35^a* displayed an altered expression response to R light in *phyQ* plants (*RS41*: WT D vs. WT R log_2_(FC) = 0.18, *phyQ* D vs. *phyQ* R log_2_(FC) = 0.52; *U2AF^35^a*: WT D vs. WT R log_2_(FC) = 1.34, *phyQ* D vs. *phyQ* R log_2_(FC) = 0.81) (Supplementary Figure S8b,c). All these changes in the expression of splicing factors might contribute to some extent to the overall alterations in the splicing landscape triggered by R-light treatment.

### 3.4. GO Term Enrichment Analysis of Light-Regulated DEGs and DSGs

**Figure 5:**
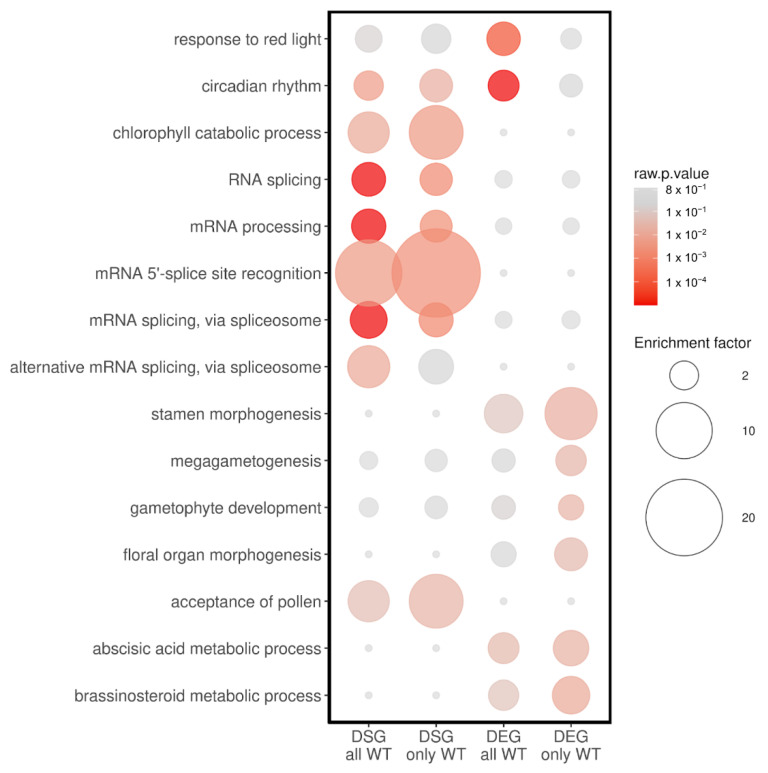
Analysis of gene ontology (GO) terms. Selected GO terms enriched among differentially spliced genes (DSGs) and differentially expressed genes (DEGs) identified in WT plants and DSGs and DEGs identified in WT but not in *phyQ* mutant seedlings are shown. The color gradient represents *p* values, and the differences in bubble size correlate with the enrichment factor.

To gain deeper insights into the genes undergoing alterations in their splicing patterns and/or expression levels, we conducted a GO term enrichment analysis. This analysis aimed to identify terms specifically associated with light pulses that are either dependent on or independent of phytochromes. We performed GO term analyses for all DEGs and DSGs under WT conditions. Additionally, we conducted separate analyses for DEGs and DSGs exclusively present in the WT but not in the *phyQ* mutant (Figure 5; Supplementary Tables S6–S9).

As expected, the category Response to R Light (GO:0010114) was prominent. Closely linked to this response, we observed enrichment in terms associated with circadian rhythms (GO:0007623). Moreover, chlorophyll levels undergo cyclic fluctuations throughout the day [45]. The perturbation of the circadian clock induced by acute light exposure during the nighttime may contribute to the enrichment of the term chlorophyll catabolic process (GO:0015996).

Consistent with prior findings, we detected a significant enrichment in terms related to splicing processes (GO:0008380, GO:0006397, GO:0000395, GO:0000398, and GO:0000380). This enrichment underscores the intricate regulation of AS, demonstrating its role in governing the splicing process itself.

Intriguingly, experiments involving night-break treatments have been shown to accelerate flowering. This phenomenon might be responsible for the enrichment of terms such as stamen morphogenesis (GO:0048448), megagametogenesis (GO:0009561), gametophyte development (GO:0048229), floral organ morphogenesis (GO:0048444), and acceptance of pollen (GO:0060321).

Furthermore, the regulation of abscisic acid (ABA) (GO:0009687) and brassinosteroid metabolic processes (GO:0016131) is intricately modulated by phyB and R-light signaling pathways [46,47], thereby imparting a broader context to our findings.

Altogether, this analysis suggests that the physiological processes regulated by phytochromes are not only a consequence of changes in mRNA expression levels but also of changes in the AS landscape.

### 3.5. Meta-Analysis of Transcriptomic Data on R-Light-Regulated AS

**Figure 6:**
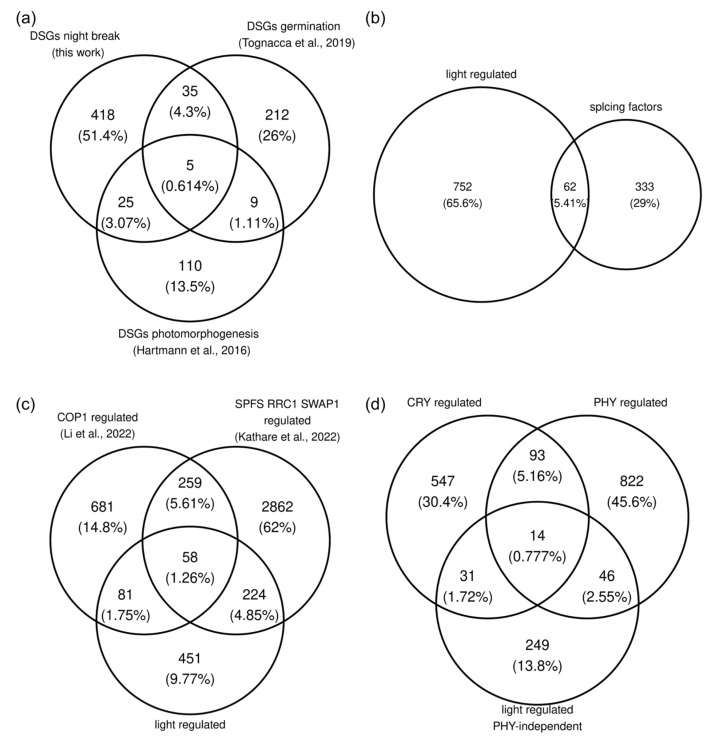
Comparison of splicing patterns in multiple datasets. (**a**) Overlap between differentially spliced genes (DSGs) upon red (R) light treatment in seeds [48], etiolated seedlings [6], and light-grown plants (this work). Both the seed and etiolated seedlings datasets were reanalyzed with the same filters used in this work. (**b**) Overlap between the union of DSGs in (**a**) and splicing regulatory factors. The list of splicing regulatory factors was taken from [3]. (**c**) Overlap between DSGs in (**a**) and DSGs regulated by COP1 (WT vs. *cop1-4*) or the splicing factors SPFS-RRC1-SWAP1 (WT vs. *spfs rrc1 swap1*) upon R-light treatment. The lists of DSGs were taken from [22,50]. (**d**) Overlap between genes regulated at the splicing level by cryptochromes, phytochromes, and chloroplasts (PHY-independent). CRY-regulated genes were obtained from the union of DSGs in WT but not in the *cry1 cry2* double mutant upon blue-light treatment in the dataset by Wang et al. (2016) [51] and Zhao et al. (2022) [5]. PHY-regulated genes were obtained from the union of DSGs between WT and the *phyA phyB* double mutant reported by [3], and the DSGs between WT and *phyB* and WT and *phyA phyB* were obtained from the reanalysis of the dataset of Wu et al. (2019) [47]. The DSGs in the *phyQ* mutant (Figure 1c) were labeled PHY-independent.

To frame our results in the current corpus of data on light-regulated splicing in Arabidopsis, we undertook a comprehensive meta-analysis of R-light-regulated splicing events across distinct developmental stages (Supplementary Table S10). We took advantage of previous datasets where R-light treatments were given to seeds to initiate germination and to etiolated seedlings to promote photomorphogenesis [6,48]. In these two datasets, together with the night-break experiment conducted here, there were a total of 814 DGSs upon R-light treatment, but only 74 of those (i.e., less than 10%) were altered simultaneously in response to the light treatment at two or more developmental stages (Figure 6a; Supplementary Table S11). This suggests that each developmental stage has a particular subset of genes susceptible to AS. It is worth noting that the differences in Figure 6a do not imply that a night-break experiment provokes more changes in splicing than the start of photomorphogenesis. The differences may simply arise from differences in the sequencing depth and read length [49].

Two out of the five DSGs in all three datasets were splicing factors: *SPF30* (AT2G02570) and *SCL30A* (AT3G13570). Indeed, many transcripts encoding splicing factors show regulated AS events [52]. Among the 814 R-light-regulated genes, there were 62 splicing factors, i.e., at least 15% of all splicing factors present in the genome (Figure 6b). Approximately half of these events were regulated by R light independent of phytochromes (Supplementary Figure S9). These results support the hypothesis that mRNAs encoding regulatory auxiliary splicing factors undergo AS upon light exposure, which in turn modulates genome-wide AS patterns under prolonged light exposure [52].

Then, we wondered how many of the 814 genes displaying R-light-regulated AS were also regulated by proteins that interact with phytochromes, particularly the splicing factors SPFS, RRC1, and SWAP1 and the ubiquitin ligase COP1, which was recently shown to be involved in the splicing regulatory network [50]. We used previously published datasets in which WT plants were compared against the triple mutant *spfs rrc1 swap1* and *cop1-4* [22,50]. Several of the genes regulated by the trio SPFS RRC1 SWAP1 were also regulated by COP1 (Figure 6c). This suggests a potential coordinated effort among phytochromes, COP1, SPFS, RRC1, and SWAP1 in orchestrating the splicing of a subset of transcripts.

Finally, we explored the different pathways through which light controls splicing. We defined light-regulated PHY-independent genes as those that were differentially spliced in response to R light in the *pyhQ* mutant (Figure 1c). Almost one-third of these genes were regulated by SPFS, RRC1, and SWAP1, suggesting a role for these splicing factors downstream of the phytochrome-independent light signaling pathway controlling AS (Supplementary Figure S10). Phytochromes are the only known sensory photoreceptors capable of absorbing R light, so we assumed that these events were likely to be regulated through some sort of retrograde signal from the chloroplast and/or by sugars generated through the photosynthetic process [53]. Interestingly, some AS events regulated by R light independent of phytochromes in light-grown plants are regulated by phytochromes and cryptochromes during de-etiolation (Figure 6d), suggesting that multiple pathways can converge on the AS regulation of the same genes but have a differential relative impact depending on the developmental stage evaluated. Overall, these results reveal the complexity of the regulatory network mediating light effects on AS, where each developmental stage presents a unique subset of light-regulated AS targets and multiple light signaling pathways regulating the splicing landscape in both redundant and non-redundant manners.

## 4. Discussion

Light is a critical source of energy and information for plants. Light perception by chlorophyll molecules triggers the photosynthetic process, which allows plants to use light as a source of energy for biomass accumulation. In turn, light perceived by phytochrome sensory photoreceptors controls multiple aspects of the plant life cycle, such as seed germination, de-etiolation, shade avoidance responses, and floral transition. Since light simultaneously excites both photosynthetic and photosensory photoreceptors, determining the relative contribution of each pathway to the control of gene expression in whole plants is challenging.

Here, we used *phyQ* mutants to disentangle the dual effect of light on AS. By exposing *phyQ* mutants to a 2-h R-light pulse given in the middle of the night, we could evaluate to what extent the effect of R light on AS was mediated by photosynthetic versus photosensory photoreceptors. Indeed, we found that phytochromes have a meaningful yet not essential contribution to AS in light-grown Arabidopsis plants. Although the regulation of some AS events depended on phytochromes, many AS events showed similar changes in splicing patterns in response to R light in both WT and *phyQ* mutants (Figure 2 and Figure 3).

The duration of the light treatment influences the specific differential splicing events that can be detected [6]. The balance between the rate of synthesis of the new isoforms and the rate of degradation of the old isoforms determines the pool of isoforms that can be quantified. In the literature, many different durations of light treatments have been applied, and this can potentially explain the discrepancies between the DSGs found in different datasets. Here, we used a 2-h light-pulse treatment to evaluate acute rather than long-term indirect responses to R light while maximizing the number of AS events that can be detected.

We found that 340 genes were susceptible to changes in AS upon R-light treatment in a phytochrome-independent manner (Figure 1c). Presumably, these splicing events are controlled by some signal derived from the photosynthetic process [7]. Indeed, light and sucrose regulate AS in a similar fashion in etiolated seedlings [6], and the effect of sugars and light on the AS of a subset of specific genes has been shown to depend on the activity of TOR in both etiolated seedlings and roots of light-grown plants [9,54]. Multiple splicing factors are phosphorylation targets of TOR, suggesting that this could be one mechanism for the regulation of AS by light acting through photosynthetic photoreceptors [55]. Additionally, light regulates AS in light-grown plants by controlling the elongation rate of RNA Pol II, which differentially affects the recognition of weak and strong donor and acceptor splice sites [8]. To further understand how light and sugars regulate AS in plants, it would be interesting to better understand the mechanistic connections, if any, between the effects of TOR on AS and those of changes in the elongation rate of Pol II. Finally, it will be worth evaluating whether photosynthetic photoreceptors acting through TOR modulate the activity of splicing factors, including those that physically interact with phytochrome and cryptochrome photoreceptors. Indeed, we found some evidence of this, as there was a significant overlap between light-regulated AS events controlled by phytochrome-interacting splicing factors and AS events regulated by light independent of phytochromes (Supplementary Figure S10).

Accumulating evidence points to a complex and dynamic network mediating the effect of light on AS. The AS of *RS31,* for example, is light-regulated in a phyA/phyB-dependent manner in etiolated seedlings, while it does not depend on phyA and phyB in light-grown plants [3,7]. Our genome-wide analysis of R-light regulation of AS in light-grown *phyQ* mutants indeed shows that the AS of many genes whose splicing patterns are regulated by phytochromes during de-etiolation is regulated by R light independently of phytochromes in light-grown plants (Figure 6d). While we cannot exclude the possibility that some of these differences may be due to particular details of each experimental setup, such as light intensity and/or duration, it is likely that the splicing landscape at a given moment is the result of complex interactions between multiple light signaling pathways, including those associated with phytochromes, cryptochromes and signals derived from photosynthesis, acting to regulate AS in a developmental context-dependent manner.

Phytochromes adopt their Pfr active conformation in response to R light and translocate to the cell nucleus, where they interact, among different proteins, with splicing factors, such as SFPS, RRC1, and SWAP1. It is hypothesized that SWAP1, RRC1, and SFPS modulate the activity of the U2 snRNP in a phytochrome-dependent manner by a not yet clear mechanism [52]. Indeed, the loss-of-function mutants of these genes display phenotypes similar to those observed in *phyB* mutants in response to R light during de-etiolation. However, whether they have a role in other phytochrome-regulated processes, such as shade avoidance responses, has not yet been studied. Addressing this question would contribute to a better understanding of the biological relevance of the interactions between phytochromes and splicing factors, which thus far have been evaluated mostly in etiolated seedlings. It would be interesting to analyze whether those interactions and their effect on AS persist in light-grown plants.

Phytochromes also appear to modulate AS through their interactions with COP1 (Figure 6c) [50]. Under dark conditions, COP1 modulates splicing by interacting with the spliceosome, and upon R-light treatment, Pfr enters the nucleus, and COP1 translocates to the cytoplasm. Given these results, we expected AS patterns in *phyQ* under R light to resemble those of WT plants in the dark. In contrast, we found that some of the phytochrome-regulated AS events behaved in *phyQ* mutants under dark and R-light conditions similarly to WT plants under R light. These results would be compatible with an active role for the Pr form of phytochromes in the modulation of AS, although evidence about a biologically active role for the phytochrome Pr form is still controversial [56]. Therefore, the more plausible scenario is that the light- and phytochrome-regulated AS events that occurred in *phyQ* as in WT under R light may be regulated by multiple signaling pathways interacting in complex ways under light and dark conditions. In line with this, other AS events showed a halfway phenotype in *phyQ* mutants in contrast to WT plants. This suggests that phytochromes may act sometimes synergistically, sometimes in parallel, and sometimes antagonistically with other light signaling pathways to regulate splicing.

Li et al. [50] mentioned that COP1 might have a role downstream of phytochromes because there were fewer COP1-regulated AS events than phyA/phyB-regulated AS events reported by Shikata et al. [3]. However, this comparison between datasets may not be fair since the RNAseq libraries and the AS-analysis pipelines differ greatly from work to work. Both library quality and AS detection software have a tremendous impact on the number of AS events that can be detected. This obstacle in dissecting the splicing regulatory network may be overcome in the near future with the reduction in costs of long-read RNA sequencing and its consequential widespread adoption [57].

During the early stages of R-light-triggered germination, many changes in the splicing landscape of non-photosynthetic seeds are observed [48]. To an unknown extent, some of these changes are likely to be regulated by phytochromes, presumably through their direct interactions with a subset of splicing factors, as shown in etiolated seedlings. However, some of the changes in AS triggered by R light in seeds could result from some metabolic signal activated as a result of the promotion of the germination process per se. The direct effects of phytochromes on AS during germination could be disentangled from the indirect effects of light on AS resulting from the reactivation of seed metabolism by examining the AS landscape of mutants such as the loss-of-function mutant of *MOTHER-OF-FT-AND-TFL1* (*MFT*), which does not require light for germination and germinates even after an FR-light pulse that inactivates the phytochrome photoreceptors [58]. Therefore, further analysis is needed to dissect the indirect effects of phytochromes on AS during the promotion of seed germination.

## 5. Conclusions

In conclusion, we evaluated the relative contribution of photosynthetic and photosensory photoreceptors to the control of AS in light-grown plants. We found that phytochromes are needed only for the proper AS of a fraction of R-light-regulated AS events, indicating that the majority of light-regulated AS events can be controlled by photosynthetic photoreceptors in light-grown plants. In addition, our meta-analysis on light regulation of AS events throughout the life cycle of plants suggests that different pathways control overlapping as well as distinct subsets of AS events, acting through similar as well as distinct regulatory mechanisms. We still need more detailed studies to quantify the relative contribution of the different photoreceptors to the regulation of AS at different developmental stages, as well as more studies addressing the role of different splicing regulators in light control of AS. Only then will we have an integrated framework that will allow us to fully understand light-regulated AS mechanisms and their biological implications.

## Data Availability

Sequencing data have been uploaded to the BioProject collection and are available under accession number PRJNA1012728. All code used in this study is publicly available at github.com/carenod/phyQ (accessed on 9 October 2023).

## Supplementary Materials

The following supporting information can be downloaded at: https://www.mdpi.com/article/10.3390/cells12202447/s1, Figure S1: Principal component analysis of all biological replicates; Figure S2: Differentially spliced events by type of ASpli supporting evidence; Figure S3: Complete PCR electrophoresis gel of light regulated genes; Figure S4: Effect of splicing on the open reading frame of selected light-regulated genes; Figure S5: Complete PCR electrophoresis gel of phy-regulated genes; Figure S6: Effect of splicing on the open reading frame of selected phytochrome-regulated genes; Figure S7: Distribution of dPIR/PSI in each subset of differentially spliced events; Figure S8: Genes that display altered expression patterns in the *phyQ* mutant; Figure S9: Overlap between the DSGs in the *phyQ* mutant and splicing regulatory factors; Figure S10: Overlap between the DSGs in the *phyQ* mutant and DSGs regulated by the splicing factors SPFS-RRC1-SWAP1 (WT vs. *spfs rrc1 swap1*) upon R-light treatment; Table S1: Number of reads obtained by sample; Table S2: Differentially spliced events in WT plants upon a night-break treatment; Table S3: Differentially spliced events in *phyQ* mutants upon a night-break treatment; Table S4: Differentially expressed genes in WT plants upon a night-break treatment; Table S5: Differentially expressed genes in *phyQ* mutants upon a night-break treatment; Table S6: Gene Ontology terms enriched among all differentially spliced genes in WT plants upon night-break treatment; Table S7: Gene Ontology terms enriched among differentially spliced genes in WT plants but not in *phyQ* mutants upon night-break treatment; Table S8: Gene Ontology terms enriched among all differentially expressed genes in WT plants upon night-break treatment; Table S9: Gene Ontology terms enriched among differentially expressed genes in WT plants but not in *phyQ* mutants upon night-break treatment; Table S10: All RNAseq datasets re analyzed with ASpli in this work; Table S11: Differentially spliced genes (DSGs) detected after reanalysis of the datasets generated by Tognacca et al. (2019) [48], Hartman et al. (2016) [6], Wang et al. (2016) [51], Zhao et al. (2022) [5], and Wu et al. (2019) [47]; Table S12: All primers used in this work.

## Abbreviations

AS—alternative splicing; NMD—nonsense-mediated decay; IR—intron retention; ES—exon skipping; Alt5′/3′ss—alternative 5′ donor/3′ acceptor splice sites; DSE—differentially spliced event; DSG—differentially spliced gene; DEG—differentially expressed gene; R—red; FR—far red; WT—wild-type; Col-0—Columbia-0; PSI—percentage spliced-in; PIR—percentage intron retention; EF—enrichment factor; GO—gene ontology.
