# Supplementary material for: Role of Phytochromes in Red Light-Regulated Alternative Splicing in Arabidopsis thaliana: Impactful but Not Indispensable
Source: Cells. 2023 Oct 13;12(20):2447. doi: 10.3390/cells12202447 (PMC10605401; doi:10.3390/cells12202447)
Supplement: Supplementary file 1 [file cells-12-02447-s001.zip › Supplementary Figures.pdf]

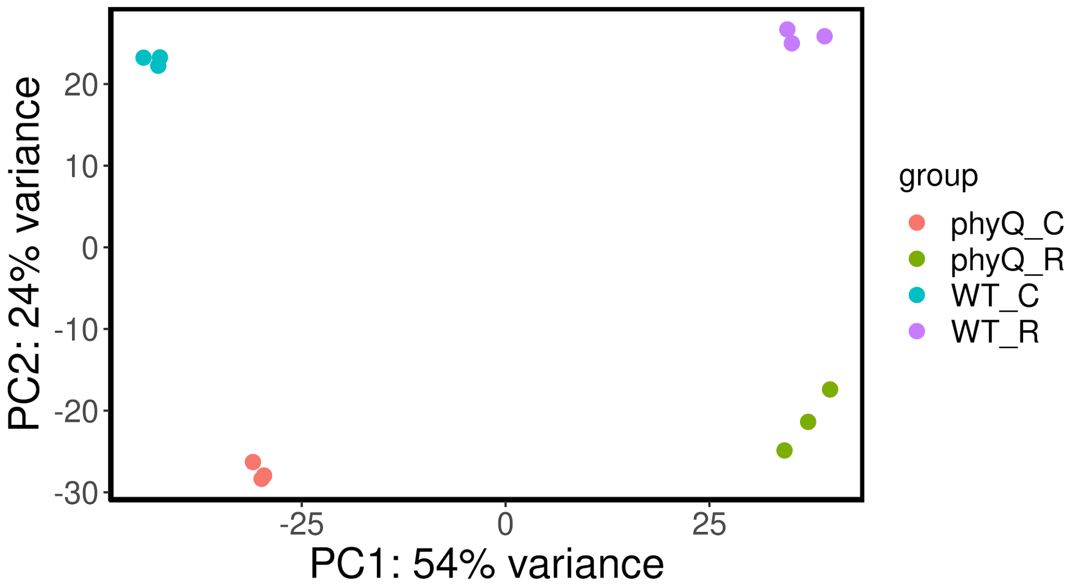

Supplementary Figure S1: Principal component analysis of all biological replicates. The analysis was performed using the normalized read counts. phyQ_C: *phyQ* Dark control; phyQ_R: *phyQ* R treatment; WT_C: wild-type (Col-0) Dark control; WT_R: wild-type (Col-0) R treatment.

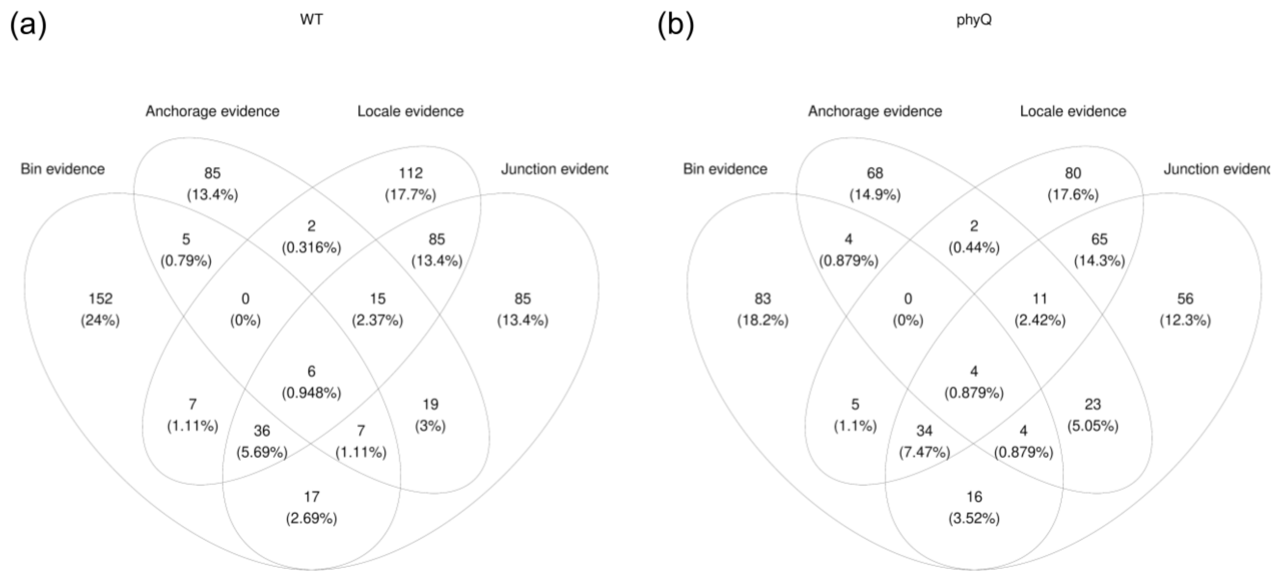

Supplementary Figure S2: Differentially spliced events by type of ASpli supporting evidence. Bin evidence refers to events where there is differential bin coverage between treatment, while anchorage, locale and junction evidence refers to events where there is differential junction use. Type of evidence for the contrasts between (a) WT dark vs WT red light and (b) *phyQ* D vs *phyQ* R light are shown.

(a) ***U2AF*^{64}**

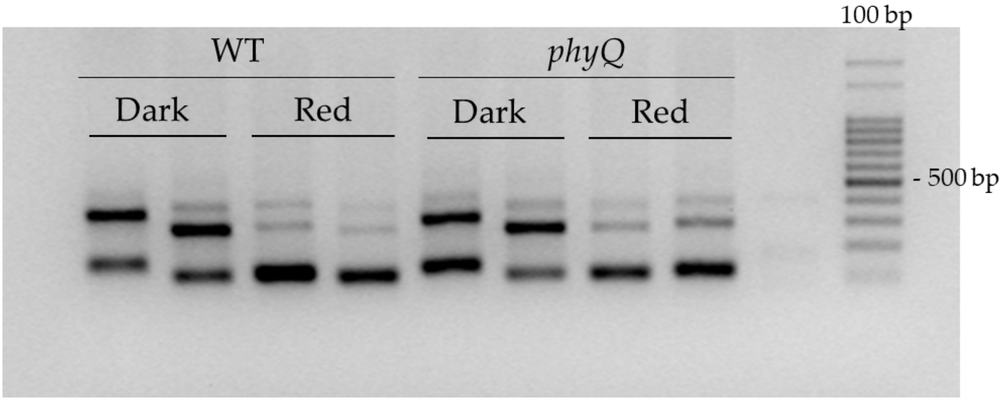

(b) ***SR30***

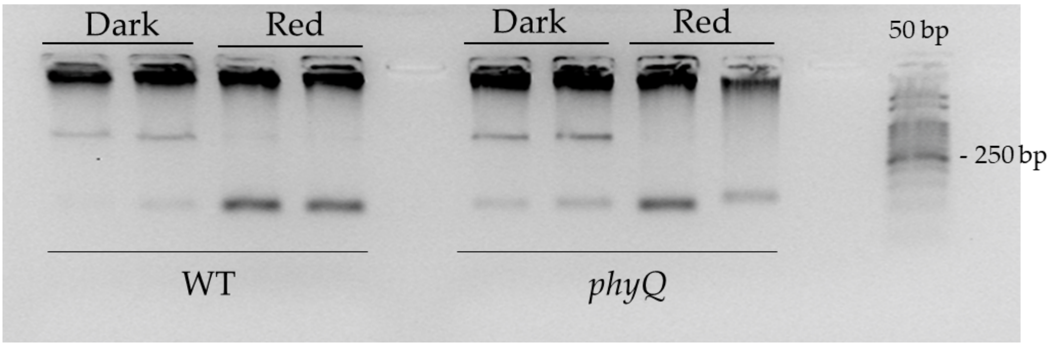

(c) ***KH29***

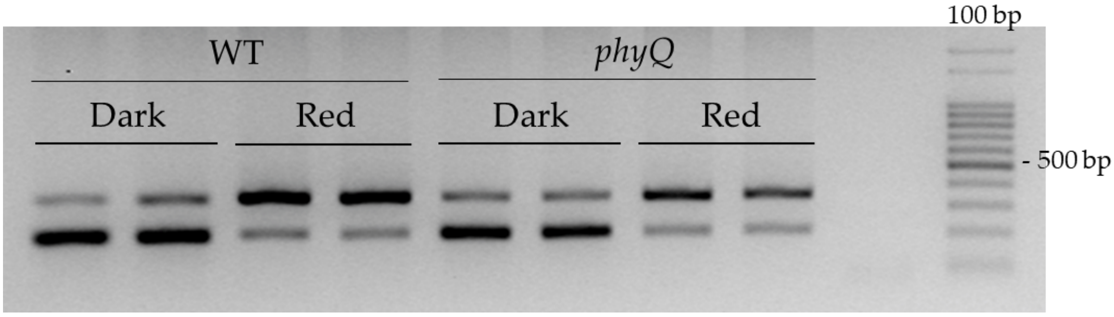

Supplementary FIgure S3: Complete PCR electrophoresis gel of light regulated genes. (a) *U2AF*^{64} (AT4G36690), (b) *SR30* (AT1G09140) and (c) *KH29* (AT5G56140). Two biological replicates are shown side by side. The order of the samples is WT dark, WT red light, *phyQ* dark and *phyQ* red light. In all cases there is a 100pb ladder.

(a) >AT4G36690 U2AF64 LONG ISOFORM

TAAAATAGTTTGGTTTCTGCATCCACCCCTTTCTTCGAATCTCGACGGCTGAAACAGATTCTAAATTTATCTCCGGTGAGAAAGAGATAGAGAGCGTTGATTCCGGCGAAAAGATG
TCTGAATTCGAAGATCACGAGGGAAACGGTACGGTTGCTGACGCGATTTACGACGAAGAGAATGGTGGCCGCGACGGCGAAATCGAGGATCAATTGGACTCGAAACCCAAGAGAGA
ATCGCGTGACCATGAAAGAGAGACATCAAGAAGTAAAGATAGAGAAAGAGAGAAGGGTCGTGATAAGGACAGAGAAAGAGACTCTGAAGTCTCTCGCCGAAGCAGGGATAGAGATG
GAGAGAAAAGCAAGGAGAGGAGTAGAGACAAAGACAGAGATCATCGTGAGCGTCATCACAGAAGTAGTCGTCACAGGGATCATAGCAGGGAGCGTGGTGAAAGAAGAGAACGTGGT
GGTAGGGATGATGATGATTACCGTCGAAGCCGTGATCGTGACCATGATAGGCGGAGAGATGATAGAGGAGGAAGACGCAGTCGTCGATCTCGATCTCGTTCAAAGGATAGATCTGA
GCGCAGAACAAGGTCACGATCTCCATCGAAAAGTAAGCAGCGAGTCAGTGGATTCGATATGGCACCTCCAGCCTCTGCAATGTTAGCTGCTGGTGCTGCTGTTACAGGCCAAGTTC
CTCCCGCTCCACCAACTCTTCCTGGGGCTGGAATGTTTCCCAACATGTTTCCATTGCCGACTGGACAGTCATTTGGGGGACTCTCTATGATGCCAATTCAGGCTATGACACAGCAG
GCAACTAGACATGCTCGCAGAGTCTATGTCGGTGGGCTTTCCCCTACGGCAAATGAACAGTCTGTGGCTACATTTTTTAGTCAAGTTATGGCTGCTGTTGGTGGAAACACTGCTGG
TCCAGGTGATGCAGTTGTTAATGTCTACATAAACCACGAGAAGAAGTTTGCTTTTGTGGAGATGCGATCTGTTGAAGAGGCAAGTAACGCTATGTCTTTGGATGGGATTATATTTG
AGGGAGCTCCGGTGAAGGTGAGGAGACCTAGTGACTATAACCCATCTCTAGCTGCGACTCTTGGTCCAAGCCAGCCCAGTCCCCATCTAAACTTAGCTGCTGTTGGTCTGACTCCA
GGTGCTTCTGGTGGGCTTGAGGGACCAGATCGTATTTTTGTTGGTGGACTTCCATATTACTTCACGGAATCACAAGTGAGGGAGTTGTTGGAGTCCTTTGGAGGCCTCAAGGGCTT
TGATCTTGTAAAAGATCGAGAAACAGGGAACTCTAAAGGATATGCCTTTTGTGTGTACCAAGATCTTTCTGTTACAGATATCGCGTGCGCTGCCCTAAATGGTATCAAGATGGGAG
ATAAGACTCTTACAGTGAGACGTGCCAACCAGGGAACAATGCTACAAAAGCCTGAACAAGAGAACGTATTATTGCATGCACAGCAGCAAATAGCTTTTCAGAGGGTTATGTTACAG
CCTGGTGCAGTGGCAACCACTGTGGTGTGTCTGACTCAAGTTGTTACTGAGGATGAGCTTAGAGATGATGAGGAGTATGGAGATATAATGGAAGACATGAGACAGGAAGGCGGAAA
GTTTGGTAAGAGACCTCTTAATTGCGCAATATGGTCTATTTTGAAATATAAGATTAAAAGCATATTGATCTGTCTCTCTGTTTTCTTGGTTGTGCTGTTTTACAGCCTTTTGCTAT
AAAGAATCAGCCCTAACATACACAGACAGGAGACTGCATAAACCTCCTAATCTATTTATCACCAATGGCCACTATTTCTTAAAAGAAAAAACTGACTTGTTTTTGTCTGTGTTTTC
TTGTCTTGTATTCGAAATGTTTTGTTCTTTAACTCTTAAAATGCAGGTGCTTTGACCAATGTTGTGATTCCGCGTCCAAGCCCCAATGGTGAGCCAGTGGCAGGCCTTGGCAAGGT
GTTTTTGAAGTATGCCGATACAGATGGCTCGACTAGGGCAAGATTCGGGATGAATGGTAGGAAATTTGGAGGAAATGAAGTCGTTGCAGTGTATTACCCTGAAGACAAGTTTGAAC
AGGGTGATTATGGAGCCTGATCACTTCTTTGTTTGCTTTCTATTACTTAAAAGTTAAGTACTTTGCTTCAGTTTCAAGACCCTTTTGCGTGATTGTAAGAGTATGAGATTATCGAC
ACGTTGCTTATTATAACGTTTTTATCTCATATCTTTGGTCTATTTCTTAAA

(b) >AT1G09140 SF30 LONG ISOFORM

AACGGTGGTTGTCTCCTTTCCAGAACAATCTAACGCTTTCTCGAACATCTTCTTCTTCTTCTTTCTCGAAATTATTTTTCCAGTAATCAATTTCTTCTCTTCTAGATTTTTACAGG
AACTAATTTTCTGCTCTGAGGTATCAGATGAGTAGCCGATGGAATCGTACGATCTACGTTGGGAATTTGCCTGGAGATATTCGCAAGTGTGAGGTTGAAGATCTCTTCTACAAGTA
TGGACCAATTGTGGACATTGATTTGAAGATTCCACCGAGACCTCCTGGTTATGCCTTTGTCGAGTTTGAAGATCCTCGTGATGCAGACGATGCAATTTATGGACGTGATGGTTATG
ATTTTGATGGGTGTCGACTTCGGGTTGAGATTGCACATGGTGGTCGTAGATTTTCACCATCAGTTGATAGGTACAGCAGCAGCTACAGTGCGAGCCGTGCACCTTCAAGACGCTCT
GACTACCGCGTGCTTGTGACCGGATTACCGCCTTCTGCTTCGTGGCAGGACCTTAAGGATCACATGCGCAAAGCTGGAGATGTCTGCTTCTCTGAAGTTTTCCCTGACCGTAAAGG
CATGTCTGGGGTTGTGGATTATAGCAACTATGATGATATGAAGTACGCAATAAGGAAACTTGATGCCACTGAATTTCGAAATGCTTTCTCTAGTGCTTATATACGGGTGAGGGAAT
ATGAGTCGAGGAGTGTGAGTCGAAGCCCAGATGATTCTAAAAGCTATAGAAGCAGGAGTCGGAGCCGTGGTCCAAGCTGTAGCTATAGTAGCAAGAGCAGGAGTGTGTCACCTGCT
AGATCCATTTCCCCGCGTTCACGGCCCCTTAGTCGTTCTCGCTCGCTATACAGCTCTGTCTCAAGATCTGGCTCACTGCTACGAGCTGGGGATTGGATCTAGATGGGTCATCTAGA
TGGATTCTTGGACTGGATTTACAAAGCTGGATTAGCATGAACTTGAACTTCTGTTTTTACGGTCTGGTCTGGTCTGGTACTCCGCGCGTATCAGCTGTAGGATCTGATCGCAAAGT
TTTGGACTATGATTACTCTGATTCCTCAATATATTTATCTTTTTGACAATAGTGGATTCTGTGTTGAGTTCTTTTCTAGGACAGCATTTAAGCTCCCGGGACTAGATGGGAGATGG
TCAGTAAATTTCTTTGTTATGCCACACTTACATGGGGTTTTTGGTCTTGCTGCAGGTCCCAATCAAGATCAAAATCAAGATCAAGATCAAGATCGAATTCTCCAGTTTCACCTGTG
ATATCTGGTTGAAAATGAAAACTGGCCACTGGCTGTACCCGAATCGTCTCAAGCTTCTCAGGCTCCACTGCTAATAGAATTTGATTCCGATTTGGGATTATTATACTGGTCTTCTT
GTATGGGACGACCAATATGTCTTTCTAGTTTTAGTTGTGAACCTGGAATTGGTCTGTTATTGTGTCATTAAAAAGCCGGAAACTCTGTCTCGGCTGCATAATAAAGTTCATCAGAC
ATTGTGTTGGGTGTGGTGAGGTTTTTCCATACATATACATTTACATTACAACTACTGGTGTCTTTTATGATTATCTTAAACTAAACTTGCAGTGTTAAAACTA

(c) >AT5G56140 KH29 SHORT ISOFORM

TAGAGAGACAACAACAGCTTTGGTTCTGCCTCCGCCGCTTCTTCTTCTTCTTCTAATCTCCGACAGATTTACTCTGATTCTGTTGTCTTCGAGATGATGATGATGTCGTCGCTTGG
TGGTGGTGGTGGTGGAGGAGGAGGAAGTGGTGGTGGTATTGGTGGTGGCGGCGGAGGGAGGTTTATGACGTATTCGTCGTCTCTTTCGGTGCCTCCTTCTGCTCCTCAGTCTCCTA
ATTACTCCGGTGGTCTCCGATCACAGTCTTCTGTCTTCGTTGAGCAAGAGAAGTATCTTTCAGAGTTACTTGCAGAGCGTCATAAGCTTACTCCATTCCTGCCTGTGCTTCCACAT
GCATTTCGGCTACTAAACCAAGAGATCTGTTCCATCATCTCCGGGACCAAATTGGCTTAACTCACCTGGTAGTTCGTCCGGTCTCATCGCCAAGCGTACAATCAGGGTCGATATTC
CAGTCGACAATTATCCGAATTTCAATTTTGTTGGTCGCCTCCTGGGTCCTAGAGGAAACTCCCTGAAGCGAGTTGAAGCAAGTACTGATTGCCGTGTTCTTATAAGAGGAAGAGGA
AGTATAAAAGATCCAATTAAGGAGGAAATGATGAGAGGAAAACCGGGATACGAGCACCTAAATGAACCACTTCATATCTTAGTGGAAGCAGAATTACCGATTGAAATAGTCGATGC
ACGGCTCATGCAAGCTCGGGAAATACTTGACGATCTTCTTACTCCTATGGAGGAAACCCATGATATGTACAAGAAACAACAGCTGCGCGAGCTAGCATTGCTCAATGGAACTCTTC
GTGAAGAAGGATCTCCAATGTCCGGTTCTGTTTCTCCTTACAATAGCCTTGGTATGAAGAGAGCCAAAACAAGAGAAGGGTAAAAACCATCGAACAGTTTGGCTGTGATCATAAAG
CAATATGAAACCGATCCAAAAGTCGACACAAAAAGGCCATACCACTCTTGACGACACGGTACTGTCTCTTGTCCTTGGGGTAGAAATGTTGTATATATGATTTATGAAATGTGTTT
GTCAGTCTATGTATCATTGTCCCGGAGAGAGATCAGATGACAAAATTGCAACGGAAACACAAGAGCTGATGAGTCTCTGTCTCTCTCCGAGACTTTGATTTTGTTTTTGTTTATGT
TTTTATGTCTGGAAAGTTGGGTTCCAACAGTTTTCTTTTAAGTTTTTTGTTTATTTGTTGTTTTAGCAAAAAGAAAACATTAAATCATGATGATGGTTTGTGTCTGGTATTGGTAA
TGATAATAATCATAATATGTTTTGGATACA

Supplementary Figure S4: Effect of splicing on the open reading frame of selected light regulated genes. 5′ UTR is colored in green, CDS in blue and 3′UTR in purple. The different shades indicate different exons. The additional sequence retained in the non-canonical AS isoform is colored white. Stop codons are shown in red and underlined.

(a) ***TTM1***

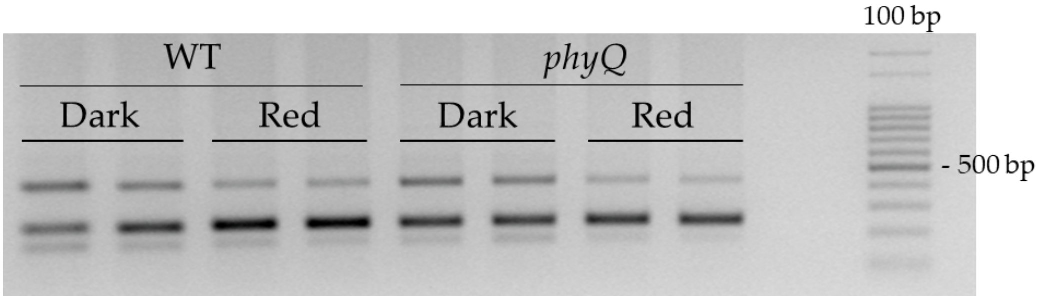

(b) ***SMD1A***

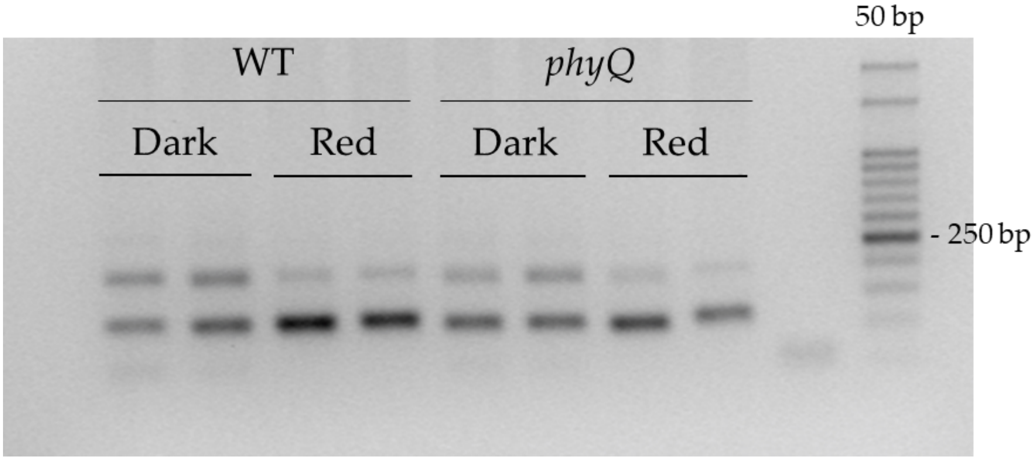

(c) **ANK1**

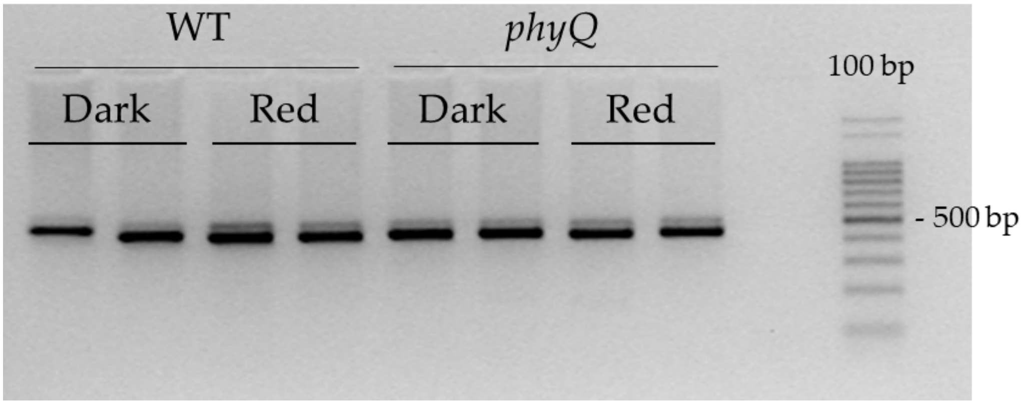

Supplementary FIgure S5: Complete PCR electrophoresis gel of phy regulated genes. (a) *TTM1* (AT1G73980), (b) *SMD1a* (AT3G07590) and (c) *ANK1* (AT5G02620). Two biological replicates are shown side by side. The order of the samples is WT dark, WT red light, *phyQ* dark and *phyQ* red light. In all cases there is a 100pb ladder.

(a) >AT1G73980 TTM1 LONG ISOFORM

GTTTACTGTTTGATTTCTCTAGTGATTTCTGGTAAAGCTTCCGTCTTCCTCCTTCCATCATCTTCTTACTCTCATTGACTGCTACGAAAATATCTCTCCAACTATATTCTTACTAT
CGCTTCTGGGTTTCCCGAAAATATGGAATAATCTCCGGCGACGTTTTTCCCCAATTTGACTCTTCTTCCGTTAGCCAAGTGTTTTAGAAGTCGTGTTGCAGTAATATTAAAGCTCA
AATCTTACATTTGGAGTGTGTGCATGTCAATTATTTCCACAGCTTCTGATGAATATTGATCGATCTTGTTAAATTGAATGAAATTTCTGCTCATGCTTTGATTTGATTAGACTTAT
ATCGTGGATTGATAGCTTCCAGATATTCTTCTTCATTTGCAACTTTGAGGCAATGGCGCTCGATAGTTCTGTTGCTCTGTCACCTCGCCGGCGGCATGGCTTGTTGCGAGATCAGG
TTCAGCTTATAAAAAGAAAGGACTCTGGAAGATATGAGATAGTTCCAATAGAGGATCCATTGTCTTTTGAAAAAGGTTTCTACGCTGTTATTCGTGCTTGTCAATTATTGGCTCAG
AAGAACGATGGGCTCATTTTGGTTGGGTTAGCTGGTCCTTCTGGAGCTGGCAAAACCATTTTCACTGAGAAGATTCTCAATTTTATGCCTAGTATTGCTATCATTAACATGGACAA
CTATAATGATGGTACTCGTGTTATCGATGGAAACTTTGATGATCCAAGGTTGACTGACTATGATACACTTCTTGACAATATACATGGTCTAAGGGATGGAAAACCTGTTCAGGTTC
CAATATATGACTTCAAGTCGAGTTCTCGAATAGGTTACAGAACGCTTGAGGTGCCTAGCTCTCGCATTGTTATTCTAGAAGGCATATATGCTTTGAGTGAGAAGCTACGGCCTTTG
CTAGATCTTCGTGTCTCTGTCACTGGTGGAGTGCATTTTGATCTTGTTAAGCGTGTTTTGCGGGACATTCAACGTGCTGGCCAGGAACCTGAAGAAATAATCCATCAGATATCTGA
GACGGTTTTCATGCTAGTCTTTGCTATCTTTTATTTTTTAGCCCAGATTAATCACCCTTGCTGTCATGCCTGCTGTTTGGAGTTGCTTCAAATATGGAAAAAACATGAAACGATGT
CTCCTACTCCTACTGGATGTTTTGAGTTTTTTTTTACAGTAAAATTTTGCTTATGTTTTTTTCTCCAACCAGGTTTATCCTATGTACAAGGCGTTTAT**TGA**ACCTGATCTGAAGAC
AGCTCAGATTAAGATCCTTAATAAGTTCAACCCATTCAGTGGCTTTCAGAACCCAACATATATTTTAAAGTCATCAAAGGCTGTAACACCCGAACAAATGAAGGCAGCTCTATCTG
AAGATTTCAAGGAACGTACAGAGGAAACTTATGACATCTATCTGTTACCACCAGGCGAGGATCCTGAAGCATGCCAATCATACCTCAGAATGAGGAACCGAGATGGAAAATACAAT
CTCATGTTTGAGGAGTGGGTTACAGATCGTCCATTTATTATATCACCCAGAATAACTTTTGAAGTTAGTGTTCGTCTTCTTGGAGGATTAATGGCACTGGGTTATACCATTGCAAC
AATCCTGAAAAGAAAGAGTCATATCTTTGATGATGACAAGGTAATTGTGAAAACTGATTGGTTGGAACAACTGAATCGGACATATGTACAGGTACAAGGTAAAGACCGTACCTTTG
TCAAAAATGTGGCAGACCAACTTGGACTGGAAGGTTCATATGTTCCACATACATATATTGAACAGATACAGCTGGAGAGGCTTGTGAATGATGTTTTGGCTTTGCCAGATGACTTA
AAAACAAAACTTAGCTTAGATGATGATACAGTTTCTAGCCCTAAAGAAGCCCTCTCAAGAGCTTCTGTTGATAGTAGAATGAAATATCTTCACGGCGGCGTATCAAAGTCTTACAC
AAACCCAAGACACAAAGTCTTGCCTAACTTGACAAGACTTGCTGTTAACAATAGAATGTTAGATGCCAGAGCCCCTGCTTCACCGGCCACTCTTCCAAATCAGGGTTTTATCACTC
AACTTTCAGACCAAATATCAACACTGAACGAGAGGATGGATGAATTCACATCCCGCATTGAAGAGTTGAATTCCAAGATCCCGAACAGGATAGCTCCTTCAGGTAGTCAACATAAC
TTGGCTTTACCAATCGAAAACGGTAATGGGTCTGTCTTATCATTCTCCGCATCCGCATCTCAGCTCGTAAGGGAATCTCCTCTGATGGAAGAGGTCGTACTGGTTGCTCGTGGACA
GCGTCAGATAATGCATCAAATGGACACACTGAGTAATCTTCTTCGGGAGTATGTTGGAGAAAAGACTCGCATAGAGAGACTCGACAGCAGCAGAACAAACAGTACAACACAAAACC
TCGAATCCTCTACTGTACCGATTCTTCTTGGTTTGGCCATTGGCTGTGTGGGCATCTTTGCCTACAGTCGTCTGAAATAGTTGCTCTAACTGTGGAAACCCAGGCTTCGTGAAATA
GTGGCAATGATGTAAATAACATGGTTTGGGATACACACTGGACAAATTCAGTCTTGACAATGTAACATGTGCCAAGAATAAAAGACTGATTCGTTTCTTACCCTTTCAATCCGGTT
CTTCTTCGTAACCAAATAAATTTAAACCGCAACACATTTAACAGGATTGACTATTTTTGGCTACTGATCAAAAAATGGAG

(b) >AT3G07590 SMD1a LONG ISOFORM

CTCTCACAGCGAAAGAAGGGTTTTGAGGGTTTTCACACCTTTCGCTGTTTCCTCGTCAATTTCTTTGTTTGGTTGTTCTTTCTTCGCATCCAAAGATGAAGCTCGTCAGGTTTTTG
ATGAAGTTAAACAATGAAACTGTGTCTATCGAGCTGAAGAATGGGACTGTGGTCCATGGAACCATCACTGGTCTTATACAAGTCGCGATCTGTTCCCTTATTTTCAATTGGCCC**TA**
ATCGCGTCCTTGTGATCACATGAATAGGTGTTGATGTGAGCATGAACACTCACTTGAAGACAGTGAAGATGAGCCTGAAGGGGAAAAACCCAGTAACACTTGATCATCTTAGCTTG
AGGGGTAATAATATTCGCTACTACATTCTTCCTGATAGCTTGAACCTCGAGACTTTACTTGTGGAAGACACTCCTAGAGTTAAACCTAAGAAGCCTGTTGCTGGGAAAGCTGTTGG
ACGCGGTCGCGGACGTGGCCGTGGGCGTGGTCGCGGCAGAGGTCGTTAGATGTAGCAGCGCTCTGGAACATGTTGAAGGGATCACACGGGTAGGGATAGGAAGATGGCACACCATA
TCCATCATGAGGCCATGGTTGATAACATGGCTCAGGTTTTTGCCATGGTTGACAACATGGCTCAGGTTTCTTCTCTTCAGGTTTCTTCTCTTCGGGTTTCTTCTCTTCGGGTTTCT
TTTCTTCTTCTGGCTTTTTGTCACCATCCTTAGGTGGTTCAACTTTAGCTACGCTAACAAGCTCCGCAAATGCAACTTTTTTC

(c) >AT5G02620 ANK1 SHORT ISOFORM

TGGACACTAAGGTTTGATCATGTGTTGTTTGTTGTATTAAGATAGTTTTAAGATATGTCTATGTGATATAACACAAGCAGCCAAAACCACGAGCATAGTTAAGAGGTAATAATATA
TTTTGTTGTTTCTTGTCCAGAGAATTCATGTTACTCTTGTCTGAATCTTCCTCAAATCTACTCATGTAGACCTCCCTATCTCGATCTAAATTTCCGCCAAACTTTTTTTAATCCCA
AAGATTCTTTAAACAGAATCATCATCAATCATGAAAGAGATCAAGAAGACGATGACGAAGCAGATGACGGCAAGACGTGACGACACGCCGTTACACACGGCGGTTAGAGAAGGCAA
AACGGACCTTCTTTTGGAGATGATCGGAGAACACGACGGCGTGGAGCTGAAAGAGTTGTTGGCCGAGCAAAACCAGTCCGGTGAAACGGCTTTGTACGTTGCGGCTGAGTACGGTT
ACACCGACATGGTTAAGATTCTGATGAAACACTCCGACTCTGTCTTAGCCGGAACCAAAGCCAAGAACGGCTTCGATGCCTTTCACATCGCCGCCAAAAACGGAAATCTACAGGTT
CTTGATGTGTTAATAGAGGCAAATCCAGAACTCTCATTTACATTTGATTCATCCAAAACCACGGCGCTTCATACCGCGGCTTCACAGGGTCATGGAGAGATTGTTTGTTTCTTGTT
GGATAAAGGTGTGGACTTAGCTGCTATTGCGAGAAGCAATGGTAAAACCGCTTTGCACTCTGCAGCTAGGAACGGGCACACGGTGATTGTGAAGAAGTTGATTGAAAAGAAGGCTG
GTATGGTTACAAGAGTCGACAAGAAAGGTCAAACCGCTCTTCATATGGCGGTTAAAGGACAAAACACTGAGATTGTGGATGTGTTGATGGAAGCAGATGGTTCGTTGATCAATTCC
GCGGATAATAAAGGAAACACTCCATTGCATATAGCAGTCCGTAAAAACAGAGCAGAGGTAATCGTTGATCAATCTTTTAAGTTATTCTTGTTGCAGAAGTTAGAATCTTTCTTGTA
TTCTTTGCAGATTGTTCAGACAGTTTTAAAGTATTGTGAAGTCAGTAGAGTGGCGGTTAATAAATCAGGAGAAACAGCTCTTGATATCGCTGAGAAAACCGGACTACATGAGATCG
TACCGCTTCTGCAAAAGATCGGTATGCAGAACGCAAGATCCATCAAGCCAGCTGAAAAAGTGGAACCATCAGGCTCTTCTAGGAAACTGAAAGAAACAGTTAGTGAAATAGGCCAT
GAGGTGCATACTCAGCTTGAACAAACCGGGAGAACGAGAAGAGAGATCCAAGGAATCGCTAAACGGGTCAACAAAATGCACACGGAAGGACTCAACAACGCGATAAACTCCACTAC
CCTAGTAGCCATTCTCATAGCAACCGTTGCTTTTGCAGCTATCTTCAACGTTCCAGGCCAGTACACGGACGACCCTAAAGACGTACCTCCAGGATATTCACTCGGAGAAGCAAGAG
CAGCTCCAAGACCCGAGTTCTTGATATTCGTCGTGTTTGATTCTTTCGCGCTTTTCATCTCTTTAGCGGTGGTTGTGGTTCAGACATCAGTGGTGGTTATAGAGAGGAGAGCAAAG
AAGCAAATGATGGCTATAATCAACAAGCTCATGTGGATGGCTTGCATTATGATCTCAGTAGCGTTCGTATCACTGTCTTTTGTTGTCGTGGGAGAGAAAGAGAAGCCTTTGGCGGT
TGGAGTAACCGCCATTGGAGCTTTGATCATGGTTTCAACACTAGGGACTATGTGTTATTGGGTGATTGCTAACCGGATTGAAGGATCTAAGTCATCTCCAGCTTCCATGATGTCAG
ATCCTGAGTTAGCTGATTCCAAACATAACCGGAAACTTTATGCGGTTTGATTAGAGTGAAACAGAGGATTTAGAAAATCATTAAAGAACAGAGAGATTTTGTATCCAGATAATAAA
AGAGATAACTCTTGTGTTTATAGTAGATTCTGTATATGTAACAAAAGGGAAATAGTATAAATCAATTTATAAGACTTTTACATGATAAATCATCAATGTTGACTTTGACTAGAGTC
AACAAAAATGACGATGCCTATACAAAGAACAAGG

>AT5G02620 ANK1 LONG ISOFORM

TGGACACTAAGGTTTGATCATGTGTTGTTTGTTGTATTAAGATAGTTTTAAGATATGTCTATGTGATATAACACAAGCAGCCAAAACCACGAGCATAGTTAAGAGGTAATAATATA
TTTTGTTGTTTCTTGTCCAGAGAATTCATGTTACTCTTGTCTGAATCTTCCTCAAATCTACTCATGTAGACCTCCCTATCTCGATCTAAATTTCCGCCAAACTTTTTTTAATCCCA
AAGATTCTTTAAACAGAATCATCATCAATCATGAAAGAGATCAAGAAGACGATGACGAAGCAGATGACGGCAAGACGTGACGACACGCCGTTACACACGGCGGTTAGAGAAGGCAA
AACGGACCTTCTTTTGGAGATGATCGGAGAACACGACGGCGTGGAGCTGAAAGAGTTGTTGGCCGAGCAAAACCAGTCCGGTGAAACGGCTTTGTACGTTGCGGCTGAGTACGGTT
ACACCGACATGGTTAAGATTCTGATGAAACACTCCGACTCTGTCTTAGCCGGAACCAAAGCCAAGAACGGCTTCGATGCCTTTCACATCGCCGCCAAAAACGGAAATCTACAGGTT
CTTGATGTGTTAATAGAGGCAAATCCAGAACTCTCATTTACATTTGATTCATCCAAAACCACGGCGCTTCATACCGCGGCTTCACAGGGTCATGGAGAGATTGTTTGTTTCTTGTT
GGATAAAGGTGTGGACTTAGCTGCTATTGCGAGAAGCAATGGTAAAACCGCTTTGCACTCTGCAGCTAGGAACGGGCACACGGTGATTGTGAAGAAGTTGATTGAAAAGAAGGCTG

GTATGGTTACAAGAGTCGACAAGAAAGGTCAAACCGCTCTTCATATGGCGGTTAAAGGACAAAACACTGAGATTGTGGATGTGTTGATGGAAGCAGATGGTTCGTTGATCAATTCC
GCGGATAATAAAGGAAACACTCCATTGCATATAGCAGTCCGTAAAAACAGAGCAGAGATTGTTCAGACAGTTTTAAAGTATTGTGAAGTCAGTAGAGTGGCGGTTAATAAATCAGG
AGAAACAGCTCTTGATATCGCTGAGAAAACCGGACTACATGAGATCGTACCGCTTCTGCAAAAGATCGGTATGCAGAACGCAAGATCCATCAAGCCAGCTGAAAAAGTGGAACCAT
CAGGCTCTTCTAGGAAACTGAAAGAAACAGTTAGTGAAATAGGCCATGAGGTGCATACTCAGCTTGAACAAACCGGGAGAACGAGAAGAGAGATCCAAGGAATCGCTAAACGGGTC
AACAAAATGCACACGGAAGGACTCAACAACGCGATAAACTCCACTACCCTAGTAGCCATTCTCATAGCAACCGTTGCTTTTGCAGCTATCTTCAACGTTCCAGGCCAGTACACGGA
CGACCCTAAAGACGTACCTCCAGGATATTCACTCGGAGAAGCAAGAGCAGCTCCAAGACCCGAGTTCTTGATATTCGTCGTGTTTGATTCTTTCGCGCTTTTCATCTCTTTAGCGG
TGGTTGTGGTTCAGACATCAGTGGTGGTTATAGAGAGGAGAGCAAAGAAGCAAATGATGGCTATAATCAACAAGCTCATGTGGATGGCTTGCATTATGATCTCAGTAGCGTTCGTA
TCACTGTCTTTTGTTGTCGTGGGAGAGAAAGAGAAGCCTTTGGCGGTTGGAGTAACCGCCATTGGAGCTTTGATCATGGTTTCAACACTAGGGACTATGTGTTATTGGGTGATTGC
TAACCGGATTGAAGGATCTAAGTCATCTCCAGCTTCCATGATGTCAGATCCTGAGTTAGCTGATTCCAAACATAACCGGAAACTTTATGCGGTTTGATTAGAGTGAAACAGAGGAT
TTAGAAAATCATTAAAGAACAGAGAGATTTTGTATCCAGATAATAAAAGAGATAACTCTTGTGTTTATAGTAGATTCTGTATATGTAACAAAAGGGAAATAGTATAAATCAATTTA
TAAGACTTTTACATGATAAATCATCAATGTTGACTTTGACTAGAGTCAACAAAAATGACGATGCCTATACAAAGAACAAGG

Supplementary Figure S6: Effect of splicing on the open reading frame of selected phytochrome-regulated genes. Effect of splicing on the open reading frame of selected light regulated genes. 5′ UTR is colored in green, CDS in blue and 3′UTR in purple. The different shades indicate different exons. The additional sequence retained in the non-canonical AS isoform is colored white. Stop codons are shown in red and underlined.

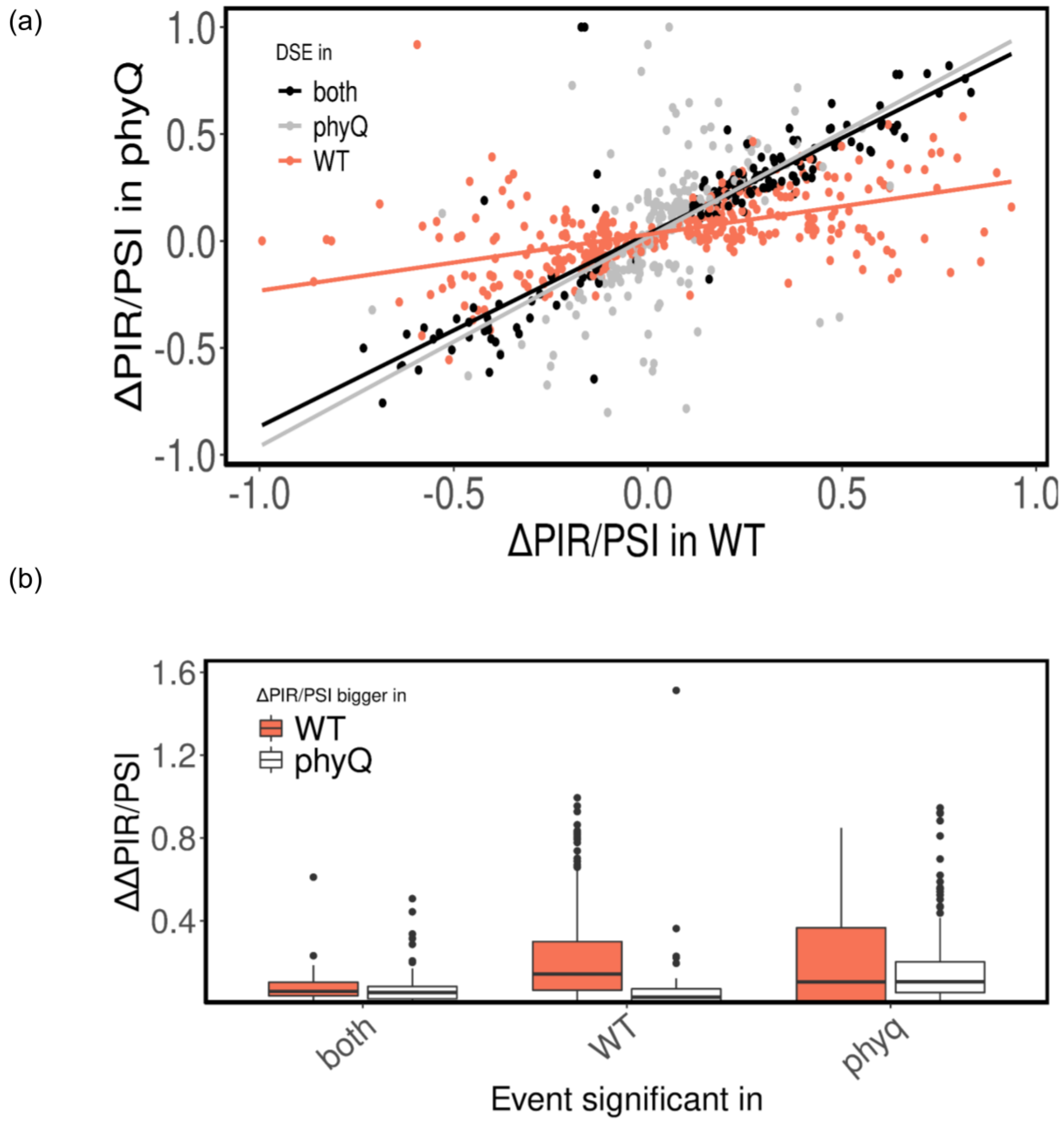

Supplementary Figure S7: Distribution of dPIR/PSI in each subset of differentially spliced events. (a) Relationship between ΔPIR/PSI values from the contrasts WT dark vs WT red light (x axis) and *phyQ* dark vs *phyQ* red light (y axis). Events are colored black if they were differentially spliced in both contrasts, orange if they were differentially spliced only in the WT contrast, and gray if they were differentially spliced only in the *phyQ* contrast. The trend lines resulting from a linear regression over the three subsets of DSEs are shown. (b) Boxplot of ΔΔPIR/PSI calculated as $|\Delta PIR/PSI_{WT} - \Delta PIR/PSI_{phyQ}|$ for DSEs in both contrasts (both), only in the contrast WT dark vs WT red light (WT), and only in the contrast *phyQ* dark vs *phyQ* red light (phyq) (x axis). In each category, the events are grouped depending on the contrast with the biggest ΔPIR/PSI. When $\Delta PIR/PSI_{WT} > \Delta PIR/PSI_{phyQ}$, events are grouped in the orange box, and when $\Delta PIR/PSI_{WT} < \Delta PIR/PSI_{phyQ}$, events are grouped in the white box.

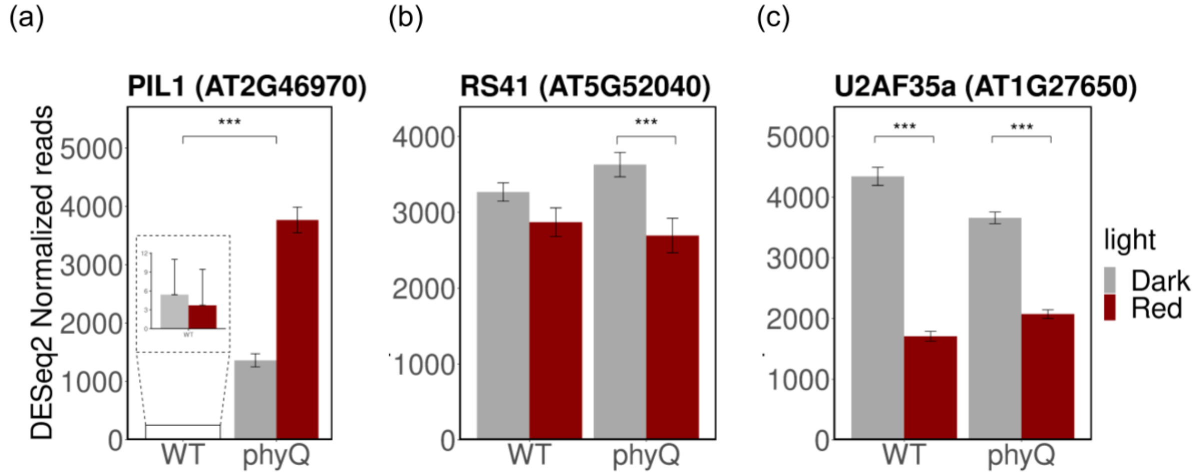

Supplementary Figure S8: Genes that display altered expression patterns in the *phyQ* mutant. Normalized read count values of (a) *PIL1* (AT2G46970), (b) *U2AF^{35}a* (AT1G27650) and (c) *RS41* (AT5G52040). The mean value and standard deviation of three independent biological replicates is shown. ***: FDR<0.001.

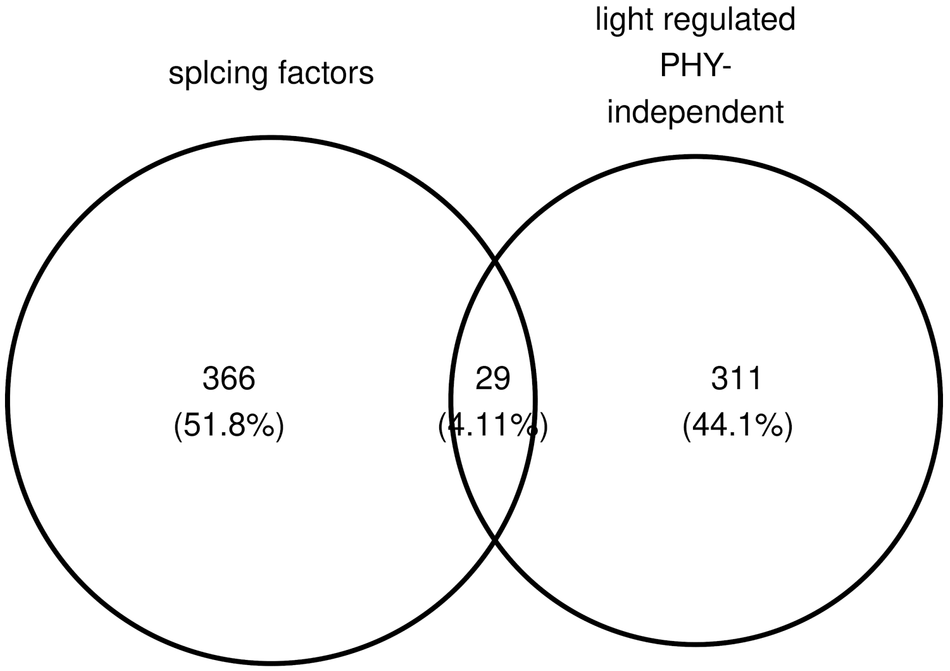

Supplementary Figure S9: Overlap between the DSGs in the *phyQ* mutant and splicing regulatory factors. The list of splicing regulatory factors was taken from Shikata *et al.* (2014). The light regulated phy-independent genes were defined as the DSEs between *phyQ* Dark and *phyQ* red light (Figure 1C).

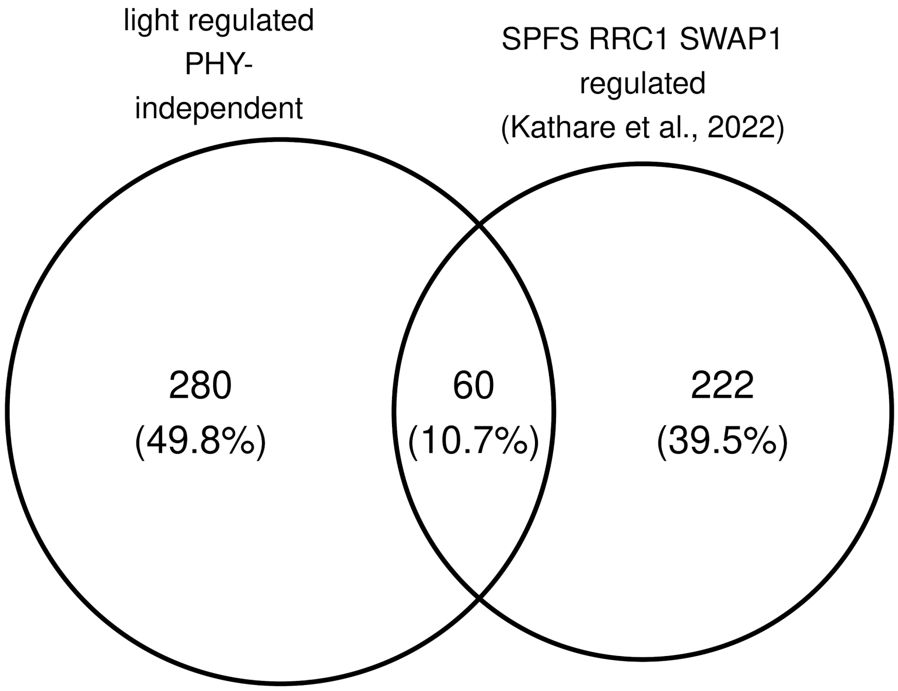

Supplementary Figure S10: Overlap between the DSGs in the *phyQ* mutant and DSGs regulated by the splicing factors SPFS-RRC1-SWAP1 (WT vs *spfs rrc1 swap1*) upon R light treatment. The lists of DSGs were taken from Kathare *et al.* (2022). The light regulated phy-independent genes were defined as the DSEs between *phyQ* Dark and *phyQ* red light (Figure 1C).
